# Development and Evaluation of a Smartphone-Based Chatbot Coach to Facilitate a Balanced Lifestyle in Individuals With Headaches (BalanceUP App): Randomized Controlled Trial

**DOI:** 10.2196/50132

**Published:** 2024-01-24

**Authors:** Sandra Ulrich, Andreas R Gantenbein, Viktor Zuber, Agnes Von Wyl, Tobias Kowatsch, Hansjörg Künzli

**Affiliations:** 1 School of Applied Psychology Zurich University of Applied Sciences Zurich Switzerland; 2 Pain and Research Unit ZURZACH Care Bad Zurzach Switzerland; 3 Department of Neurology University Hospital Zurich Zurich Switzerland; 4 Institute for Implementation Science in Health Care University of Zurich Zurich Switzerland; 5 School of Medicine University of St.Gallen St. Gallen Switzerland; 6 Centre for Digital Health Interventions Department of Management, Technology, and Economics ETH Zurich Zurich Switzerland

**Keywords:** chatbot, mobile health, mHealth, smartphone, headache management, psychoeducation, behavior change, stress management, mental well-being, lifestyle, mindfulness, relaxation, mobile phone

## Abstract

**Background:**

Primary headaches, including migraine and tension-type headaches, are widespread and have a social, physical, mental, and economic impact. Among the key components of treatment are behavior interventions such as lifestyle modification. Scalable conversational agents (CAs) have the potential to deliver behavior interventions at a low threshold. To our knowledge, there is no evidence of behavioral interventions delivered by CAs for the treatment of headaches.

**Objective:**

This study has 2 aims. The first aim was to develop and test a smartphone-based coaching intervention (BalanceUP) for people experiencing frequent headaches, delivered by a CA and designed to improve mental well-being using various behavior change techniques. The second aim was to evaluate the effectiveness of BalanceUP by comparing the intervention and waitlist control groups and assess the engagement and acceptance of participants using BalanceUP.

**Methods:**

In an unblinded randomized controlled trial, adults with frequent headaches were recruited on the web and in collaboration with experts and allocated to either a CA intervention (BalanceUP) or a control condition. The effects of the treatment on changes in the primary outcome of the study, that is, mental well-being (as measured by the Patient Health Questionnaire Anxiety and Depression Scale), and secondary outcomes (eg, psychosomatic symptoms, stress, headache-related self-efficacy, intention to change behavior, presenteeism and absenteeism, and pain coping) were analyzed using linear mixed models and Cohen *d.* Primary and secondary outcomes were self-assessed before and after the intervention, and acceptance was assessed after the intervention. Engagement was measured during the intervention using self-reports and usage data.

**Results:**

A total of 198 participants (mean age 38.7, SD 12.14 y; n=172, 86.9% women) participated in the study (intervention group: n=110; waitlist control group: n=88). After the intervention, the intention-to-treat analysis revealed evidence for improved well-being (treatment: β estimate=–3.28, 95% CI –5.07 to –1.48) with moderate between-group effects (Cohen *d*=–0.66, 95% CI –0.99 to –0.33) in favor of the intervention group. We also found evidence of reduced somatic symptoms, perceived stress, and absenteeism and presenteeism, as well as improved headache management self-efficacy, application of behavior change techniques, and pain coping skills, with effects ranging from medium to large (Cohen *d*=0.43-1.05). Overall, 64.8% (118/182) of the participants used coaching as intended by engaging throughout the coaching and completing the outro.

**Conclusions:**

BalanceUP was well accepted, and the results suggest that coaching delivered by a CA can be effective in reducing the burden of people who experience headaches by improving their well-being.

**Trial Registration:**

German Clinical Trials Register DRKS00017422; https://trialsearch.who.int/Trial2.aspx?TrialID=DRKS00017422

## Introduction

### Background

Primary headaches, including tension-type headaches (TTHs) and migraine, are among the most prevalent neurological illnesses [1]. TTH and migraine are ranked as the third and sixth most common diseases, respectively, worldwide in both women and men [2]. The physical, social, and mental burden of headaches, defined as the summation of all negative consequences [3], is substantial. In addition, it is important to consider the quality of life impacted by headaches, defined as the subjective assessment of general well-being, position, and prospects in life. Individuals who have recurrent headaches are often afraid of the next headache attack, which can lead to avoidance behaviors, such as the cancelation of social activities or not even planning them [4].

The economic costs of headaches are substantial, primarily manifesting as indirect and intangible costs. This includes work absences because of headaches (absenteeism), reduced on-the-job performance while experiencing headaches (presenteeism), reduced quality of life, and increased pain outside the workplace [5-7]. In the European Union, the total annual costs associated with headaches among adults are estimated at €173 billion (US $189 billion; 64% migraine, 12% TTH, and 24% other types of headaches) [8]. A more recent study conducted in Canada estimated the total annual cost associated with migraine to be CAD $23,756.04 (US $17,750.50) per patient [9]. Given the high personal and financial costs, there is an urgent need for effective management of headaches.

Headaches are multifactorial, and besides physiological factors, lifestyle factors, such as stress or sleep, play a significant role in the development and retention of a headache [10-12]. Insufficient perception of stress reactions, individual attitudes toward stress (eg, high-performance orientation and anxiety), or coping strategies, such as avoidance versus endurance, are seen as dysfunctional stress-coping in relation to headaches [13,14]. The belief that pain-related factors are outside one’s control and the perceived inability to control these factors (ie, low self-efficacy) are further dysfunctional coping mechanisms associated with poor adjustment to headache and psychological functioning [15,16]. Among people who have headache, stress, stress regulation, and mental tension are perceived as critical triggers of a headache [14,17-19], and headache itself serves as a stressor that negatively affects well-being [20]. However, there are controversial findings regarding the association between lifestyle and headache, and individuals differ in the extent to which these factors interact with headache [21].

Guidelines recommend pharmacological and nonpharmacological interventions as standard therapy [22], including behavioral treatment, which has been shown to be effective in both face-to-face and web-based settings [22-25]. These treatments incorporate psychoeducation, relaxation techniques, physical activity, triggers management, and cognitive behavioral therapy elements, focusing on stress management and coping strategies to modify negative and dysfunctional cognitions, emotions, and behavior related to headaches [26,27]. A person-centered approach that integrates various intervention components is more effective [28,29] and may enhance personal control and efficacy in headache management [14]. However, challenges such as cost, access, motivation [18], and stigma hinder engagement [30]. Despite this, consistent care using nonmedical options has proven beneficial [31].

In addition to traditional evidence-based treatment modalities, mindfulness-based interventions have gained research interest, showing enhanced well-being in various settings [32-34], particularly for coping with chronic conditions, such as chronic pain [35-37]. Studies have demonstrated that mindfulness interventions benefit individuals with headaches by improving psychological functioning [38-40]. Furthermore, they help improve affective conditions, such as anxiety and depression, which are often related to poorer treatment outcomes [41,42]. Improvements in these conditions, even if subclinical, may improve coping skills for headaches and increase treatment adherence. In addition, individuals without a psychiatric diagnosis may experience headache-attributed disability, defined as physical, cognitive, and mental incapacities imposed by headaches [3], such as disabling anxiety related to fear of headaches and perceived triggers [43].

Over the past few years, the adoption of app-based interventions for headache management has increased [44,45]. These interventions primarily take the form of electronic headache diary apps, which offer practical solutions for data monitoring [46]. Furthermore, app-based interventions have improved our understanding of the relationship between lifestyle factors and headaches [21]. Apps are also suitable for providing guideline-compliant therapeutic options [23], such as psychoeducation, relaxation techniques, endurance sports, and other elements of behavioral therapy (eg, stress reduction). However, evidence of the effectiveness of app-based behavioral interventions for managing headaches remains weak [44,47].

Conversational agents (CAs), also known as chatbots or digital assistants, are increasingly being applied in both clinical [48-50] and nonclinical [51-53] health care settings to support disease management and behavioral lifestyle interventions. CAs engage users in humanlike conversations [48,54-56], enabling factual, relational, and emotional communication. This interactive style enhances engagement by establishing a working alliance between the users and the CAs [57,58], reflecting the collaborative relationship between shared treatment goals and tasks [59], which is crucial for treatment success in psychotherapy and counseling [60]. In contrast, conventional mobile health (mHealth) interventions may lack a therapist relationship and suffer from noncommittal timing [61], may not be used as intended [62], and engagement may often be low [63-65]. Nevertheless, CA-based coaching offers the potential to deliver personalized, accessible, and scalable content via web-based or mobile-based apps [56,66].

### Objectives

To our knowledge, there is no evidence of mHealth coaching interventions delivered by CAs for the treatment of headaches. Building on a successful pilot study [67], we designed BalanceUP, a smartphone-based and CA-delivered intervention aimed at supporting a healthy lifestyle in people with headache. BalanceUP aims to improve mental well-being by promoting behavior change techniques (BCTs) in behaviors, emotions, thoughts, and beliefs related to headaches while ensuring low-threshold access and scalability. Using smartphone apps’ technical potential, scalable interventions can be beneficial in supporting individuals [68]. Consequently, this study had the following objectives: (1) to develop a smartphone-based and CA-delivered intervention for people with headache and (2) to evaluate its effectiveness, engagement, and acceptance.

## Methods

### App Development

**Figure 1:**
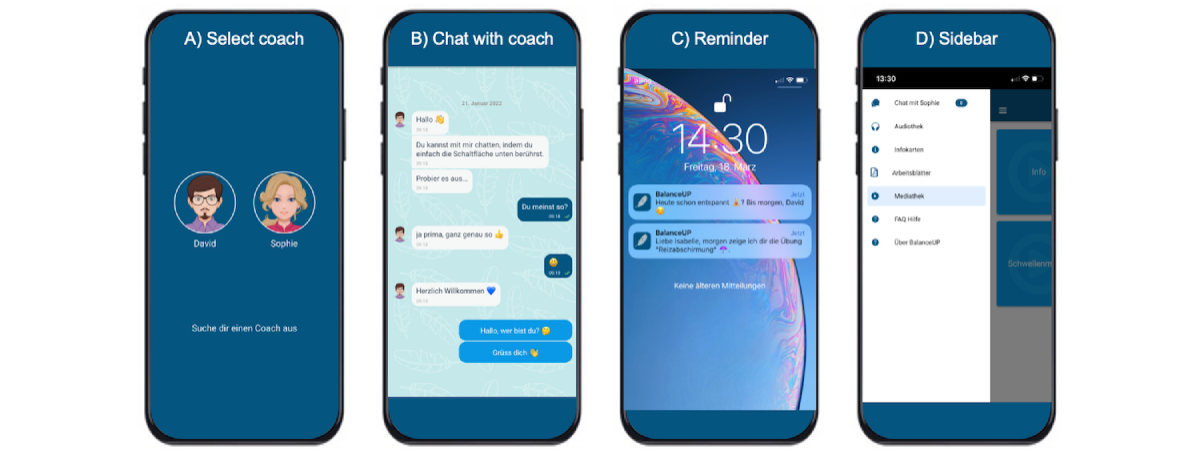
Screenshots of the BalanceUP app: (A) conversational agent (CA) selection screen, (B) screen displaying a chat with the CA, (C) screen displaying push reminder from the CA, and (D) sidebar of the app.

BalanceUP, developed for iOS (Apple Inc) and Android (Google LLC) platforms using MobileCoach [54,69,70], provides a chat-based interface for communicating with the CA (Figure 1B). The communication between users and the server is encrypted. The chat feature offers predefined answer options and free-text input, guiding conversations along dynamic paths to individualized tasks. The CA also shares videos and pictures elaborating on the psychoeducational content. Using the sidebar, users can access the (1) chat channel; (2) audio library (eg, relaxation, mindfulness, and imagination exercises); (3) illustrations; (4) working materials (eg, energy balance and coping circle); (5) video library (eg, animated psychoeducational videos); and (6) frequently asked questions about the study and BalanceUP app (Figure 1D).

### Coaching Intervention

#### Overview

**Figure 2:**
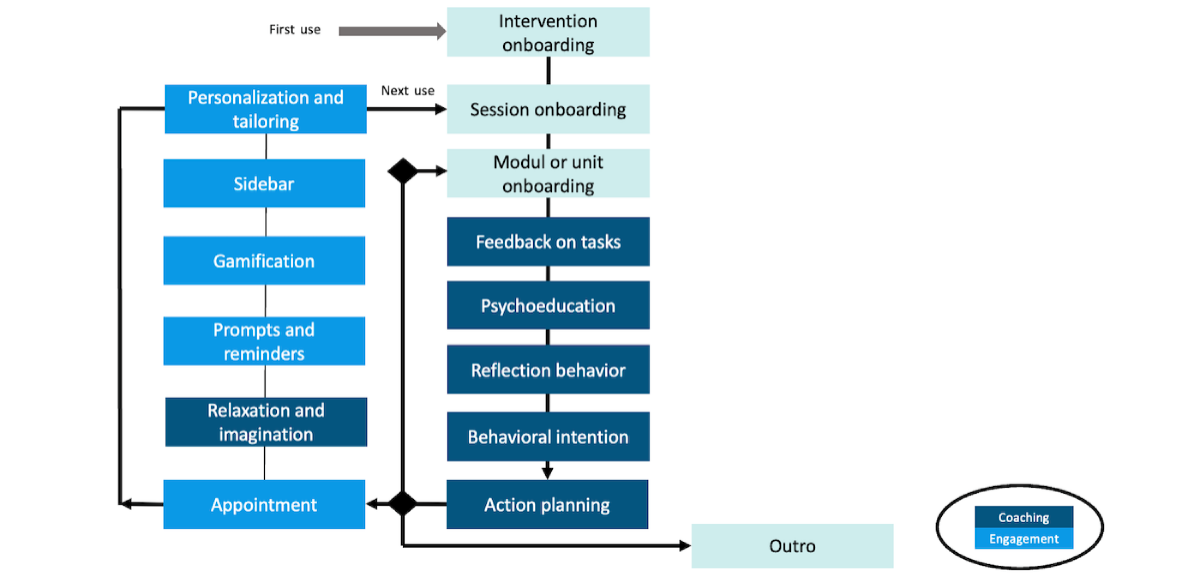
Elements of coaching and engagement.

**Figure 3:**
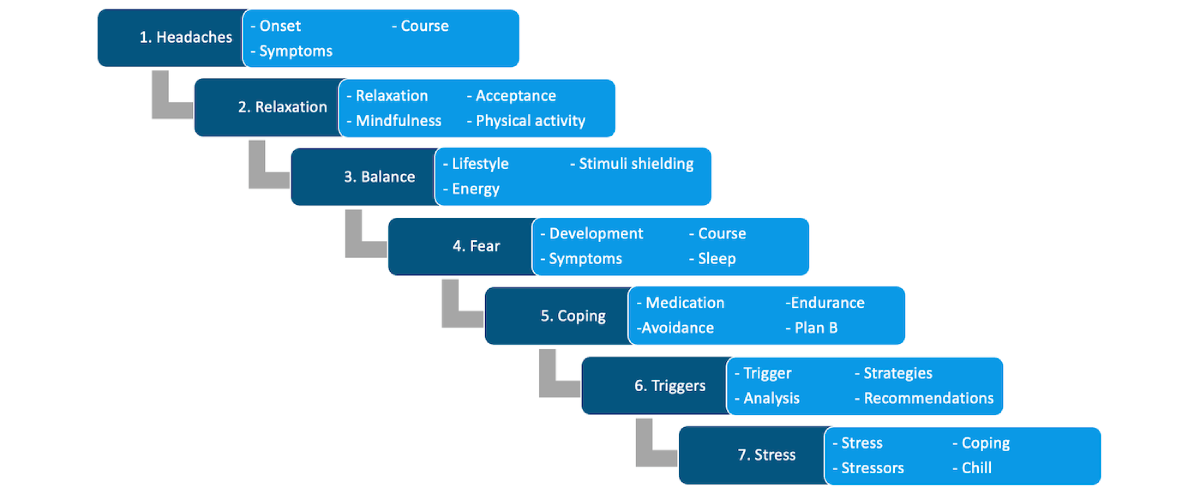
Structure of modules and units.

**Figure 4:**
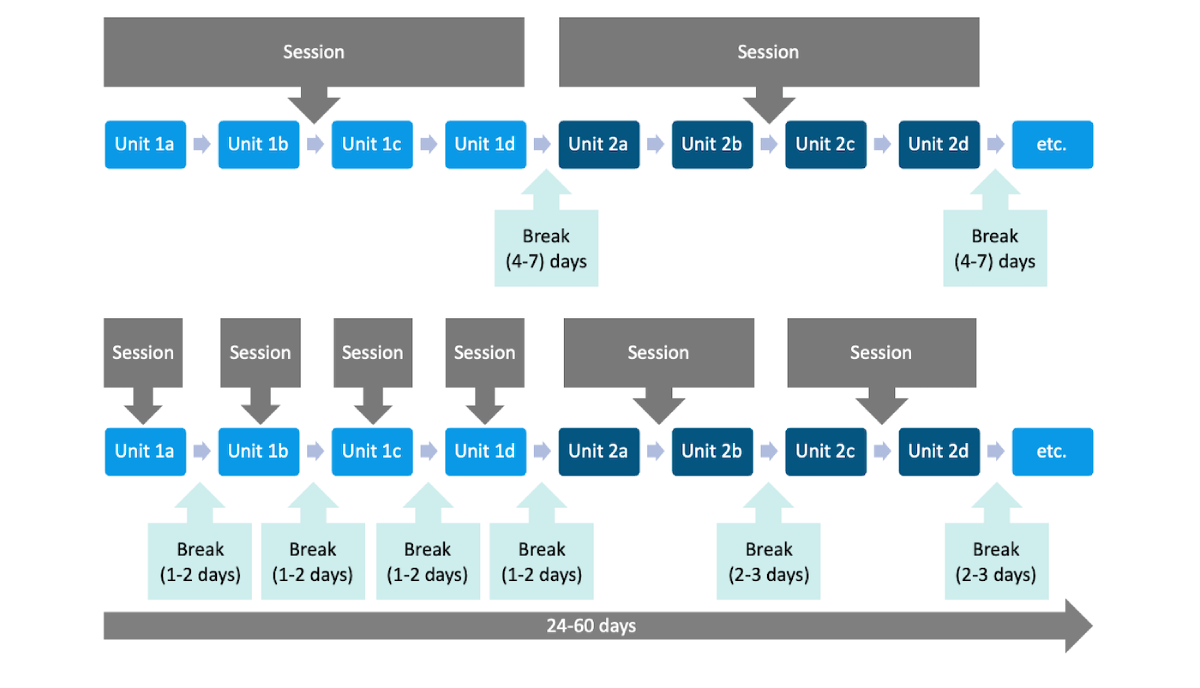
Examples of session flow.

Drawing on the best practice from behavior therapy, BalanceUP is based on the cognitive behavior change migraine therapy manual (Cognitive-Behavioral Therapy for Migraine Management [Kognitiv-verhaltenstherapeutisches Migränemanagement]; MIMA) [13], which has been demonstrated to be feasible [71] and effective, showing results similar to those of the active control group at a 12-month follow-up [72]. BalanceUP comprises 7 consecutive modules: (1) headaches, (2) relaxation, (3) balance, (4) fear, (5) coping, (6) trigger, and (7) stress (refer to Multimedia Appendix 1 for an overview of the coaching), with a procedure similar to MIMA: (1) feedback on tasks, (2) psychoeducation, (3) reflection of behavior, (4) behavioral intention, (5) action planning, and (6) relaxation and imagination (refer to Figure 2 for further details). Each module contains 3 to 4 units (Figure 3), and users can work through these units during sessions with the CA according to their preferences, allowing completion within 24 to 60 days. Examples of the session flow can be found in Figure 4. After completion, users can still access the materials. A diagram showing the intervention flow is presented in Multimedia Appendix 2.

#### Behavior Change

To foster health-promoting behaviors, various BCTs [73] have been considered (eg, gamification for reward, action planning, and prompts to perform an exercise). A complete list of BCTs and their specific applications is outlined in Multimedia Appendix 3 [73]. BCTs are intervention ingredients designed to alter or redirect causal processes that regulate behavior [74]. Their specific implementation enables accurate replication, precise specification of the intervention content, and investigation of possible mechanisms of action [74].

#### Engagement

Tailoring (at a subgroup level) and personalization (at an individual level) are essential for promoting trust, engagement, adherence, and effectiveness in mHealth interventions [63,75]. Both tailoring (eg, psychoeducation material based on headache types) and personalization (eg, coach selection [Figure 1A] personal greeting, personalized goals, and individual appointments with the CA) were applied in BalanceUP. Reminders (Figure 1C) are an effective way of improving engagement, especially when they address specific needs [76,77]; refer to Multimedia Appendix 4 [49,56,68,78-81] for the detailed aspects of the engagements implemented.

#### Intended Use

The intended use of BalanceUP, which is the extent to which an individual needs to experience the content to derive maximum benefit from the intervention [81], is determined by reaching the outro and completing the postsurvey. Users can skip a module or elements based on their diagnosis (migraine vs TTH) and preferences. For instance, participants can skip psychoeducation within a unit and proceed to behavior reflection, whereas relaxation exercises, videos, and worksheets are optional. This procedure offers flexibility in line with the recommended mHealth intervention strategies [82,83] and is similar to the dose-response rate in pharmaceutical research, where medication dosage can vary depending on the patient’s condition and characteristics. Furthermore, we base this approach on the self-determination theory [84], which emphasizes autonomy, competence, and engagement.

### Study Design and Procedure

**Figure 5:**
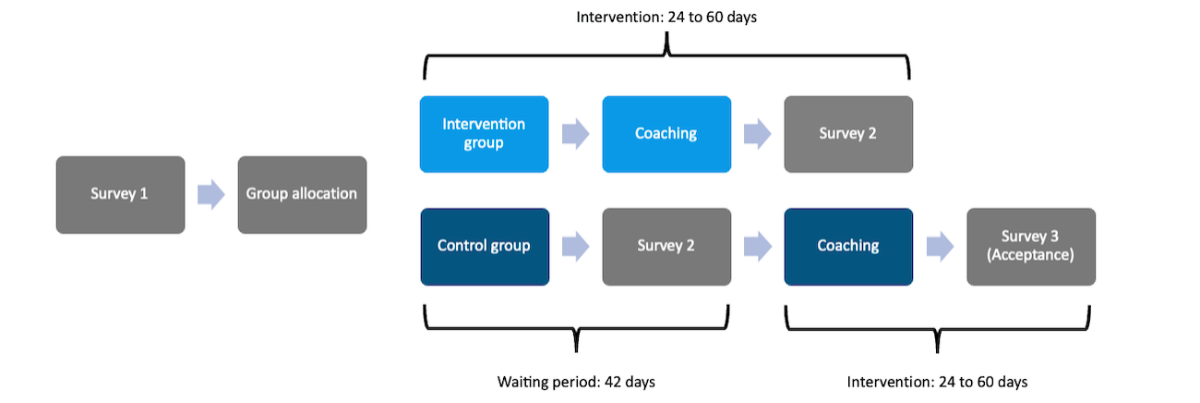
Study procedure.

We conducted an unblinded, 2-arm, randomized controlled trial (RCT). After onboarding, eligible participants were asked to provide electronic informed consent. Participants who provided consent proceeded to complete the baseline survey (T1), while those who were ineligible were directed to a farewell conversation with the CA. Those meeting the inclusion criteria and consenting were randomly assigned with a 1:1 allocation ratio to either the intervention or waitlist control group using random numbers (0-1) generated by the BalanceUP app, with numbers below 0.5 assigned to the intervention group. The intervention group immediately began with the coaching. The postintervention survey (T2) was conducted 24 to 60 days after randomization. The waitlist control group received weekly reminders from the CA during the 42-day waiting period. After this, they completed the postintervention survey (T2) and were given the option to access coaching or proceed to the outro (Figure 5). Throughout the study, self-reported primary and secondary outcomes were collected within the BalanceUP app and via the in-app survey tool, LimeSurvey (version 3.4). Refer to Multimedia Appendix 5 for outcome details and data collection times.

### Study Participants

Participants were self-recruited in Switzerland, Germany, and Austria between April and November 2022 (German-speaking parts) via the study website. The link to the website was shared via social media by headache organizations, insurance companies, and health care institutions. This recruitment strategy allowed participation from all German-speaking countries. The study website provided study information and app download links. Inclusion criteria were being an adult (aged *≥*18 y) with a smartphone (eg, iOS [Apple Inc] or Android [Google LLC]), fluent German-speaking skills, and experiencing regular headaches for at least 3 months with a minimum of 4 incidents per month. These criteria were assessed using the BalanceUP app during onboarding.

### Outcomes

#### Mental Well-Being

To measure effectiveness, we defined mental well-being as the primary outcome, measured by the Patient Health Questionnaire Anxiety and Depression Scale (PHQ-ADS) [85]. The PHQ-ADS is a composite of the Patient Health Questionnaire-9 (PHQ-9) [86] and General Anxiety Disorder Scale-7 (GAD-7) [87]. Scores can range from 0 to 48, with higher scores indicating more severe depression and anxiety; 3 to 4 points were considered the minimum clinically important difference. Cutoff scores of 10, 20, and 30 denoted mild, moderate, and severe degrees of depression and anxiety, respectively.

To assess psychological functioning, multiple secondary outcome measures were used in accordance with the established guidelines [88].

#### Secondary Outcomes

##### Depression

The PHQ-9 [86] consists of 9 items for evaluating depressive symptoms, rated on a 4-point Likert scale ranging from 0 (not at all) to 3 (nearly every day). Higher scores indicate higher symptom severity, with scores ranging from 0 to 4 indicating no symptoms of depression and scores from 5 to 9, 10 to 14, 15 to 19, and 20 to 27 indicating mild, moderate, moderately severe, and severe depression, respectively.

##### Anxiety

The GAD-7 [87] is used for evaluating symptoms of generalized anxiety disorder. It comprises 7 items. Similar to PHQ-9, answers are rated on a 4-item Likert scale ranging from 0 (not at all) to 3 (nearly every day). Higher scores indicate higher levels of anxiety, and the total score ranges from 0 to 21. Scores from 0 to 4, 5 to 9, 10 to 14, and 15 to 21 denoted minimal, mild, moderate, and severe anxiety, respectively.

##### Somatic Symptoms

We measured somatic symptoms using the Patient Health Questionnaire-15 (PHQ-15) [89]. It is a 15-item self-report questionnaire that can be scored on a scale from 0 (not impaired) to 2 (severely impaired). A total score of ≥15 on the PHQ-15 indicates a high level of impairment owing to somatic symptoms [90]. For this study, we adopted 2 items from the PHQ-9 because the items were similar. However, the answer scale of the 2 items differed between the PHQ-9 and PHQ-15 and had to be converted according to the manual.

##### Stress

We measured stress with the German version of the Perceived Stress Scale-10 [91]. The 10-item questionnaire can be rated on a scale from 0 (never) to 4 (very often); higher scores reflect a higher level of perceived stress, and scores can range from 0 to 40.

##### Self-Efficacy

To assess headache-related self-efficacy, we used the German short form of the Headache Management Self-Efficacy (HMSE-G-SF) Scale [92]. The measurement consists of 6 items assessing self-efficacy beliefs related to headaches. Answer scales range from 1 (do not agree) to 7 (agree). Higher scores implied higher self-efficacy expectations, and summed scores <19 indicated below-average self-efficacy expectations compared with other people experiencing headache.

##### Intention to Change Behavior

To assess participants’ intention to change behavior, we used the health action process approach model [93], which categorizes behavior change into 3 stages: nonintenders, intenders, and actors. In this study, participants indicated their use of psychological techniques for headache treatment by choosing 1 of the 5 possible answers: (1) no, and I do not intend to do so (nonintender); (2) no, but I am considering it (nonintender); (3) no, but I have the intention to do so (intender); (4) yes, but it is not easy (actor); and (5) yes, and it is easy (actor).

##### Absenteeism and Presenteeism

To measure work-related impairment due to headaches, we applied 4 out of 5 questions from the Migraine Disability Assessment [94]. These questions assessed days with complete loss and days with at least 50% reduced productivity (eg, work, household, and school) for the past 3 months. Given the study’s runtime and potential recall challenge [94], participants reported headache days for a 1-month period instead of 3 months.

##### Pain Processing

We applied the Questionnaire for the Assessment of Pain Processing (questionnaire to assess pain management; Fragebogen zur Erfassung der Schmerzverarbeitung) to measure pain coping strategies [95]. This tool assesses coping strategies in individuals with persistent pain and is comprised of 2 parts. In this study, we used the first part to evaluate cognitive and behavioral coping using 24 items. The cognitive coping subscale included the dimensions “action planning skills,” “cognitive restructuring,” and “experience of competence.” The behavioral coping subscale included “mental distraction,” “counteracting activities,” and “rest and relaxation techniques.” Answers were scored from 1 (not at all true) to 6 (always true), with higher scores indicating better pain processing.

##### Sociodemographics

We collected data on age, sex, level of education, parallel app use for headache tracking, concurrent psychotherapy, commitment to the program, and headache diagnosis at baseline to describe the study population. We further assessed participants’ sensitivity to triggers and tendency to avoid triggers optionally in module 6 (Trigger) using the German short version of the Headache Triggers Sensitivity and Avoidance Questionnaire [96]. Mean scores <2.03 indicate below-average trigger sensitivity and scores >3.19 indicate above-average trigger sensitivity, respectively. For avoidance, mean scores <2.09 indicate below-average trigger avoidance and scores >3.23 indicate above-average trigger avoidance, respectively.

#### Engagement

##### Overview

According to a systematic review conceptualizing engagement with digital behavior change interventions [64], engagement is both a multidimensional concept and a dynamic process. Engagement consists of 2 parts: (1) the extent of use (eg, amount, frequency, duration, and depth) and (2) a subjective experience characterized by attention, interest, and affect. By using this multidimensional approach, we aimed to capture the various aspects of engagement, as defined by Perski et al [64].

##### Extent of Use

The following use data were recorded during the coaching intervention: total minutes spent on in-app relaxation and imagination exercises, total reminders sent to participants in cases of inactivity, and the average number of days taken to complete 1 coaching module. In addition, the percentage of answered conversational turns between the participant and the CA coach was calculated, where a higher number indicated higher engagement with the intervention. We also assessed intended use, that is, the number of participants who completed the outro.

##### Subjective Experience

To gather the subjective experiences of the participants, we used 4 items from the German Group Therapy Session Evaluation by Patients [97] with statements about personal involvement, active participation, perceived comprehensibility, and perceived benefit on a 5-point Likert scale ranging from 1 (disagree) to 5 (agree). Furthermore, we measured perceived enjoyment by applying a single-item measure from technology acceptance research [98,99] (“Did you enjoy the last unit?”), ranging from 1 (not at all) to 5 (very much).

We assessed participants’ commitment to changing their behavior with 1 question (“How committed are you towards changing your behavior?”) on a scale of 1 to 10.

We used a modified version of the Session Alliance Inventory—Patient Version [100,101] to repeatedly measure the working alliance between the participant and the CA. It consists of 3 items for the Bond Scale and 3 items for the Task and Goal Scale. In this study, we used the items of the validated German version of the Working Alliance Inventory-Short Revised [102], which features a 5-point answer scale ranging from 1 (seldom) to 5 (always). In addition, we contextualized the Session Alliance inventory by replacing the term “therapist” with the CA’s name.

#### Acceptance

We assessed the acceptance of BalanceUP with a slightly modified and translated version of the Mobile App Rating Scale (uMARS) [103] assessing engagement (eg, entertainment, interest, customization, interactivity, and target group of the app); information (eg, quality of information, quantity of information, visual information, and credibility of source); perceived quality (eg, recommendation, use, payment, and overall rating); and perceived impact (eg, awareness, knowledge, attitudes, behavior change, seeking help, and intention to change). All subscales used a 5-point Likert scale ranging from 1 to 5, with higher scores indicating a more favorable judgment.

In addition to the uMARS, the questions “What did you like most about the BalanceUP app?” and “What would you like to see improved about the BalanceUP app?” could be answered in free text.

#### Impression of Change and Adverse Events

We assessed adverse events with the Patients Global Impression of Change Scale [104]. It is a 7-point scale depicting the perceived overall improvement in general health, rated from 1 (much improved) to 7 (very much worse). In this study, for scores ≥5, we also assessed whether participants believed these changes occurred because of coaching or whether other circumstances (eg, professional situation and conflicts in the social sphere) caused these changes. Participants were asked to note the adverse changes if coaching was given as a reason.

### Sample Size Calculation

We estimated the sample size based on the primary outcome (mental well-being) measured by the PHQ-ADS for a linear mixed model (LMM) and a repeated measure ANOVA (within-between interaction). Consistent with previous headache and chronic pain research [105-107], we assumed a small-to-medium effect size for the primary outcome. Statistical power calculation using G*Power^3^ software revealed that a sample size of 90 (45 for each group) would be sufficient, with a power of 0.80 to detect a small-to-medium time×group interaction effect size (Cohen *d*=0.35) with an α of .05 and based on 2 measurements. According to our pilot study, we estimated a dropout rate of 40% and aimed to recruit approximately 150 participants.

### Data Analysis

The analysis was performed using SPSS (version 28.0; IBM Corp) and R software (version 4.2.2; R Foundation for Statistical Computing) including the *lme4*. For the primary outcome (PHQ-ADS) from before the intervention (T1) to after the intervention (T2), the LMMs were used, considering time (T1 and T2), group (intervene and wait), and their interaction as fixed effects, with participants as a random factor. Secondary outcomes (ie, PHQ-9, GAD-7, PHQ-15, Perceived Stress Scale-10, Questionnaire for the Assessment of Pain Processing, HMSE-G-SF, Migraine Disability Assessment, and health action process approach) were analyzed accordingly. Missing data were managed using LMM, which is based on all observed data and accounts for data missing at random [108-110]. According to the CONSORT (Consolidated Standards of Reporting Trials) guidelines, we reported the LMM analysis for the (1) intention-to-treat (ITT) analysis, in which all randomized participants were included, regardless of whether they used the coaching and (2) per-protocol (PP) analysis of complete cases. Calculations of within- and between-group effect sizes (Cohen *d*) were based on the pooled SD of complete cases only and labeled as small (Cohen *d*=0.2), medium (Cohen *d*=0.5), and large (Cohen *d*=0.8). The influence of predictors on outcomes was explored using the LMM, including the primary outcome (PHQ-ADS), with a focus on the 3-way interaction. Change in engagement over time was analyzed via repeated-measures ANOVA, and the effect of early engagement [64] on treatment outcomes was analyzed using linear regression. Descriptive statistics were used to summarize participant characteristics at baseline, and 2-tailed *t* tests were used to assess baseline differences. We applied qualitative content analysis [111,112] to answer the open-ended questions.

### Ethical Considerations

The Swiss Ethics Committee Zurich reviewed the research project and confirmed (Swiss Ethics BASEC-Nr. Req-2021-01365) that it does not fall within the scope of the Human Research Act. This research project was registered in the World Health Organization–accredited German Clinical Trials Register (DRKS00017422). We performed this trial based on the CONSORT-EHEALTH guidelines.

## Results

### Participant Flow and Baseline Characteristics

**Figure 6:**
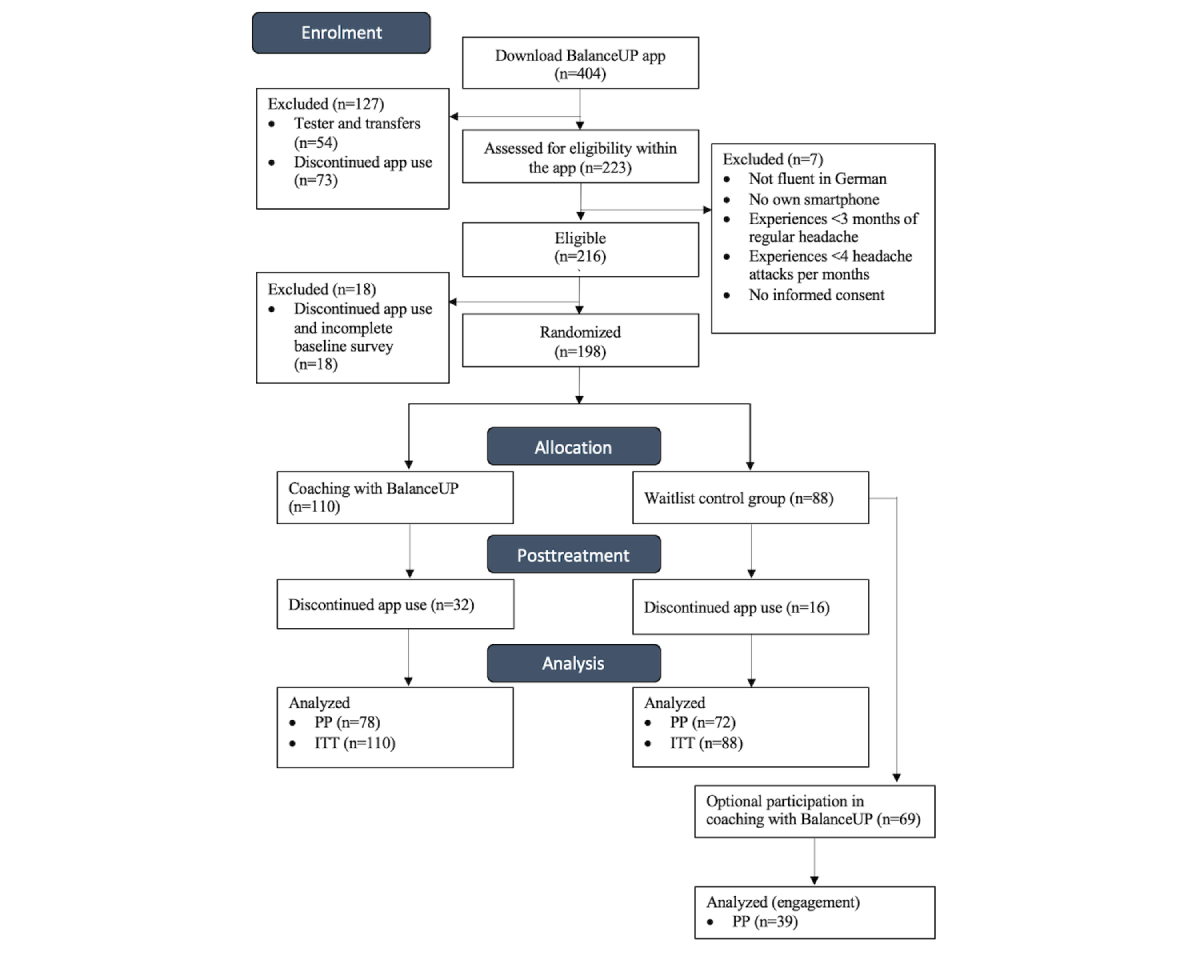
Participant flowchart. ITT: intention-to-treat; PP: per-protocol.

During the recruitment phase, from April to November 2020, 405 individuals downloaded the BalanceUP app. Of these, 223 (55.1%) were assessed for eligibility and 7 (1.7%) were excluded from the study. Of those eligible, 198 (48.9%) individuals completed the baseline survey and were randomized into the intervention (n=110) and control (n=88) groups. The full participant flow is presented in Figure 6. The dropout rate for randomized participants after the treatment was 29.1% (32/110) for the intervention group and 18% (16/88) for the waitlist control group and thus can be considered low, particularly for a fully unguided mHealth intervention [113] and in comparison with our pilot study.

As presented in Table 1, most of the participants were women (180/198, 90.9%) with a mean age of 38.7 (SD 12.14) years, and more than half had a university degree (104/198, 52.5%). Migraine was the most prevalent diagnosis, accounting for 72.2% (143/198) of the sample. Approximately half of the participants (95/198, 48%) reported using headache diaries to track their symptoms. Few participants (32/198, 16.2%) reported attending psychotherapy and using the coaching app. In general, participants reported an average of 6.66 (SD 7.34) days of work per month missed because of headaches and 11.82 (SD 9.77) days per month when their performance was reduced by half or more (including work, school, and household). Compared with other individuals who had headaches [92], the study participants reported average levels of headache-related self-efficacy. On average, participants were classified as “intenders,” indicating that they had the intention to change their behavior (as opposed to “nonintenders” or “actors”). On average, the participants had mild depression (mean 9.06, SD 4.24), mild anxiety (mean 6.76, SD 3.87), and moderate psychosomatic symptoms (mean 10.92, SD 4.34). There was no difference between the groups in any of the outcomes.

**Table 1:** Demographic, app-related, headache-related, and mental well-being–related characteristics at baseline (N=198).

				Control group (n=88)		Intervention group (n=110)		*P* value^a^	
**Demographic characteristics**									
	Age (y), mean (SD)			38.28 (12.82)		39.03 (11.46)		.67	
	**Gender, n (%)**								.64
		Man	8 (9.1)		12 (10.9)				
		Woman	76 (86.4)		96 (87.3)				
		Nonbinary	1 (1.1)		1 (0.9)				
		No information	3 (3.4)		1 (0.9)				
	**Education, n (%)**								.22
		No education	2 (2.3)		0 (0)				
		Obligatory or high school	1 (1.1)		6 (5.5)				
		Vocational training and high school	26 (29.5)		34 (30.9)				
		Higher vocational training	13 (14.8)		12 (10.9)				
		University or University of Applied Sciences	46 (52.3)		58 (52.7)				
**App related, n (%)**									
	**Platform**								.09
		iOS (Apple Inc)	53 (60.2)		53 (48.2)				
		Android (Google LLC)	35 (39.8)		57 (51.8)				
	**Chatbot coach**								.62
		Sophie (woman)	76 (86.4)		94 (85.6)				
		David (man)	12 (13.6)		16 (14.5)				
**Headache-related characteristics**									
	**Diagnosis, n (%)**								.82
		Migraine	65 (73.9)		78 (70.9)				
		TTH^b^	10 (11.4)		12 (10.9)				
		No diagnosis	13 (14.8)		20 (18.2)				
	**Tracking app in parallel, n (%)**								.82
		Yes	43 (48.9)		52 (47.3)				
		No	45 (51.1)		58 (52.7)				
	**Psychotherapy, n (%)**								.49
		Yes	16 (18.2)		16 (14.5)				
		No	72 (81.8)		94 (85.5)				
	Absenteeism: MIDAS^c^ (d), mean (SD)^d^			6.95 (8.3)		6.37 (6.49)		.60	
	Presenteeism: MIDAS^c^ (d), mean (SD)^d^			12.1 (9.99)		11.54 (9.54)		.70	
	**Pain coping: FESV^e^ score, mean (SD)**								
		Cognitive coping	41.06 (9.1)		39.52 (8.51)		.22		
		Behavioral coping	31.58 (7.55)		29.86 (7.73)		.19		
	Self-efficacy: HMSE-G-SF^f^ Scale score, mean (SD)			22.77 (6.78)		24.37 (6.94)		.11	
	Application of behavior change techniques^g^, mean (SD)			3.36 (0.97)		3.45 (1.04)		.09	
**Mental well-being, mean (SD)**									
	Depression: PHQ-9^h^ score			9.43 (4.13)		8.69 (4.34)		.22	
	Anxiety: GAD-7^i^ score			7.25 (3.86)		6.25 (3.87)		.07	
	Somatic symptoms: PHQ-15^j^ score			11.26 (4.28)		10.57 (4.4)		.27	
	Stress: PSS^k^ score			30.32 (5.75)		29.8 (6.33)		.55	

^a^Baseline group comparison between intervention and waitlist control groups using the 2-tailed *t* test and chi-square test.

^b^TTH: tension-type headache.

^c^MIDAS: Migraine Disability Assessment conducted for a period of 1 month.

^d^Waitlist control group: n=83 and intervention group: n=102 (outliers removed).

^e^FESV: questionnaire to assess pain management (Fragebogen zur Erfassung der Schmerzverarbeitung).

^f^HMSE-G-SF: German short form of the Headache Management Self-Efficacy.

^g^Application of behavior change techniques based on the health action process approach stages of change: 1 to 2=nonintenders, 3=intenders, and 4 to 5=actors.

^h^PHQ-9: Patient Health Questionnaire-9.

^i^GAD-7: General Anxiety Disorder Scale-7.

^j^PHQ-15: Patient Health Questionnaire-15.

^k^PSS: Perceived Stress Scale.

### Effectiveness

#### Primary Outcome

Table 2 presents the results of the LMM analyses for the ITT and PP analyses. For both the ITT and PP analyses, we found evidence of a treatment effect (group by time interaction) for the PHQ-ADS after the intervention (t_342_=–3.6; *P*<.001, 95% CI –5.06 to –1.47 and t_294_=–3.58; *P*<.001, 95% CI –5.11 to –1.49, respectively). BalanceUP significantly affected mental well-being, as shown by the change in the PHQ-ADS. However, the waitlist control group did not improve with time (Cohen *d*=–0.07, 95% CI –0.23 to 0.08), the intervention group improved from before the intervention to after the intervention with a medium effect (Cohen *d*=0.62, 95% CI –0.84 to –10.39).

**Table 2:** Results of per-protocol (PP) and intention-to-treat (ITT) outcome analysis using linear mixed models^a,b^.

Outcome		PP		ITT	
		β estimate (SE; 95% CI)	*P* value	β estimate (SE; 95% CI)	*P* value
**Mental well-being (PHQ-ADS)^c^**					
	Intercept	17.01 (N/A^d^)	N/A	16.68 (N/A)	N/A
	Time^e^	–0.60 (0.67; –1.91 to 0.72)	.37	–0.50 (0.66; –1.80 to 0.80)	.45
	Group^f^	–0.6 (0.38; –4.00 to 0.74)	.18	–1.75 (1.07; –3.84 to 0.35)	.10
	Treatment^g^	–3.3 (0.93; –5.12 to –1.48)	<.001	–3.28 (0.91; –5.07 to –1.48)	<.001
**Depression (PHQ-9^h^)**					
	Intercept	9.68 (N/A)	N/A	9.43 (N/A)	N/A
	Time	–0.33 (0.37; –1.06 to 0.40)	.37	–0.27 (0.36; –0.99 to 0.45)	.47
	Group	–0.80 (0.70; –2.19 to 0.59)	.26	–0.74 (0.61; –1.95 to 0.47)	.23
	Treatment	–1.78 (0.51; –2.79 to –0.77)	<.001	–1.80 (0.51; –2.79 to –0.80)	<.001
**Anxiety (GAD-7^i^)**					
	Intercept	7.33 (N/A)	N/A	7.25 (N/A)	N/A
	Time	–0.26 (0.39; –1.04 to 0.51)	.50	–0.23 (0.39; –0.99 to 0.53)	.55
	Group	–0.83 (0.60; –2.07 to 0.06)	.17	–1.01 (0.54; –2.06 to 0.06)	.06
	Treatment	–1.52 (0.54; –2.59 to –0.45)	.006	–1.45 (0.53; –2.50 to –0.40)	.007
**Somatic symptoms (PHQ-15^j^)**					
	Intercept	11.42 (N/A)	N/A	11.26 (N/A)	N/A
	Time	–4.44 (0.38; –0.75 to 0.75)	>.99	0.04 (0.38; –0.70 to 0.78)	.92
	Group	–0.66 (0.72; –2.07 to 0.75)	.36	–0.69 (0.64; –1.95 to 0.58)	.29
	Treatment	–2.33 (0.53; –3.37 to –1.30)	<.001	–2.33 (0.52; –3.35 to –1.30)	<.001
**Stress (PSS-10^k^)**					
	Intercept	30.74 (N/A)	N/A	30.32 (N/A)	N/A
	Time	–0.90 (0.62; –2.13 to 0.33)	.15	–0.76 (0.62; –1.96 to 0.45)	.22
	Group	–0.25 (1.03; –2.28 to 1.79)	.81	–0.52 (0.91; –2.31 to 1.27)	.57
	Treatment	–2.69 (0.86; –4.39 to –0.99)	.002	–2.60 (0.85; –4.26 to –0.93)	.003
**Headache management self-efficacy (HMSE-G-SF^l^Scale)**					
	Intercept	23.14 (N/A)	N/A	22.77 (N/A)	N/A
	Time	–0.07 (0.73; –1.51 to 1.37)	.92	0.08 (0.72; –1.34 to 1.50)	.91
	Group	1.37 (1.20; –0.80 to 3.52)	.26	1.60 (0.98; –0.34 to 3.54)	.11
	Treatment	4.15 (1.01; 2.15 to 6.14)	<.001	4.05 (0.99; 2.10 to 6.00)	<.001
**Application of behavior change techniques^m^ (HAPA^n^)**					
	Intercept	3.47 (N/A)	N/A	3.36 (N/A)	N/A
	Time	0.01 (0.11; –0.22 to 0.23)	.93	–0.07 (0.12; –0.16 to 0.29)	.55
	Group	0.21 (1.14; –0.08 to 0.49)	.15	0.09 (0.14; –0.18 to 0.36)	.51
	Treatment	0.70 (0.16; 0.39 to 1.01)	<.001	0.76 (0.16; 0.45 to 1.07)	<.001
**Absenteeism and presenteeism^o^ (MIDAS^p^)**					
	Intercept	20.10 (N/A)	N/A	19.05 (N/A)	N/A
	Time	–0.90 (1.17; –3.21 to 1.41)	.45	–0.68 (1.15; –2.95 to 1.59)	.56
	Group	–2.12 (2.58; –7.20 to 2.96)	.41	–1.14 (2.20; –5.46 to 3.18)	.61
	Treatment	–4.61 (1.63; –7.83 to –1.39)	.005	–4.81 (1.60; –7.97 to –1.66)	.003
**Cognitive pain coping (FESV^q^)**					
	Intercept	40.69 (N/A)	N/A	41.06 (N/A)	N/A
	Time	1.40 (0.83; –0.24 to 3.03)	.93	1.28 (0.82; –0.33 to 2.89)	.12
	Group	–1.48 (1.42; –4.23 to 1.32)	.30	–1.54 (1.26; –4.02 to 0.94)	.22
	Treatment	5.56 (1.14; 3.30 to 7.83)	<.001	5.58 (1.13; 3.37 to 7.80)	<.001
**Behavioral pain coping (FESV)**					
	Intercept	31.72 (N/A)	N/A	31.58 (N/A)	N/A
	Time	0.64 (0.71; –2.03 to 0.76)	.37	–0.59 (0.70; –1.97 to 0.79)	.40
	Group	–1.40 (1.23; –3.84 to 1.03)	.26	–1.72 (1.10; –3.89 to 0.45)	.12
	Treatment	4.91 (0.98; 2.87 to 6.84)	<.001	5.00 (0.97; 3.10 to 6.90)	<.001

^a^Outcomes of the PP analyses: only participants who completed the outro (ie, intended use).

^b^Outcomes of the ITT analyses: all participants who were randomized into groups.

^c^PHQ-ADS: Patient Health Questionnaire Anxiety and Depression Scale.

^d^N/A: not applicable.

^e^Time effect represents the rate of improvement for both intervention and waitlist control groups.

^f^Group effect represents intervention or waitlist control group.

^g^Treatment effect is represented by group and time interaction.

^h^PHQ-9: Patient Health Questionnaire-9.

^i^GAD-7: General Anxiety Disorder Scale-7.

^j^PHQ-15: Patient Health Questionnaire-15.

^k^PSS-10: Perceived Stress Scale-10.

^l^HMSE-G-SF: German short form of the Headache Management Self-Efficacy.

^m^Application of behavior change techniques, based on the health action process approach stages of change: 1 to 2=nonintenders, 3=intenders, 4 to 5=actors.

^n^HAPA: health action process approach.

^o^Assessment of absenteeism and presenteeism based on Migraine Disability Assessment, conducted for a period of 1 month.

^p^MIDAS: Migraine Disability Assessment.

^q^FESV: questionnaire to assess pain management (Fragebogen zur Erfassung der Schmerzverarbeitung).

Changes in the PHQ-ADS score differed significantly between groups with a medium effect (Cohen *d*=0.66, 95% CI –0.99 to –0.33); refer to Table 3 for observed means and effect sizes (Cohen *d*) for participants who completed the coaching intervention as intended (PP).

**Table 3:** Results of per-protocol outcome measures: means and effect sizes (Cohen *d*).

Measure			Before the intervention, mean (SD)	After the intervention, mean (SD)	Within-group effect (before the intervention vs after the intervention), Cohen *d*^a^ (95% CI)	Between-group effect (intervention vs waitlist control group), Cohen *d* (95% CI)
**Primary outcome**						
	**Mental well-being (PHQ-ADS^b^)**					
		Intervention (n=78)	15.38 (7.09)	11.49 (5.48)	–0.62 (–0.84 to –0.39)	–0.66 (–0.99 to –0.33)
		Control (n=72)	17.01 (7.36)	16.42 (9.18)	–0.07 (–0.23 to 0.08)	N/A^c^
**Secondary outcome**						
	**Depression (PHQ-9^d^)**					
		Intervention (n=78)	8.88 (4.19)	6.77 (3.55)	–0.54 (–0.74 to –0.35)	–0.59 (–0.91 to –0.26)
		Control (n=72)	9.68 (4.27)	9.35 (5.15)	–0.07 (–0.23 to 0.09)	N/A
	**Anxiety (GAD-7^e^)**					
		Intervention (n=78)	6.50 (3.64)	4.72 (2.41)	–0.58 (–0.83 to –0.32)	–0.66 (–0.98 to –0.33)
		Control (n=72)	7.33 (3.91)	7.07 (4.53)	–0.06 (–0.24 to 0.12)	N/A
	**Somatic symptoms (PHQ-15^f^)**					
		Intervention (n=78)	10.76 (4.03)	8.42 (3.66)	–0.61 (–0.82 to –0.39)	–0.65 (–0.98 to –0.32)
		Control (n=72)	11.42 (4.26)	11.42 (5.46)	0.00 (–0.15 to 0.15)	N/A
	**Stress (PSS-10^g^)**					
		Intervention (n=78)	30.49 (6.12)	26.9 (6.06)	–0.59 (–0.80 to –0.37)	–0.43 (–0.76 to –0.12)
		Control (n=71)	30.66 (5.68)	29.79 (7.34)	–0.13 (–0.32 to 0.05)	N/A
	**Headache management self-efficacy (HMSE-G-SF^h^ Scale)**					
		Intervention (n=78)	24.5 (9.79)	28.58 (7.21)	0.58 (0.34 to 0.82)	0.81 (0.48 to 1.14)
		Control (n=71)	23.14 (6.37)	23.07 (6.34)	–0.01 (–0.20 to 0.18)	N/A
	**Application of behavior change techniques^i^ (HAPA^j^)**					
		Intervention (n=78)	3.45 (0.89)	4.38 (0.52)	1.28 (0.92 to 1.63)	1.05 (0.71 to 1.39)
		Control (n=71)	3.46 (0.94)	3.48 (1.12)	0.02 (–0.20 to 0.24)	N/A
	**Absenteeism and presenteeism^k^ (MIDAS^l^)**					
		Intervention (n=71)	17.99 (14.18)	12.48 (12.05)	–0.42 (–0.60 to –0.23)	–0.45 (–0.79 to –0.12)
		Control (n=67)	20.10 (16.48)	19.21 (17.36)	–0.05 (–0.18 to 0.07)	N/A
	**Cognitive pain coping (FESV^m^)**					
		Intervention (n=78)	39.22 (7.98)	46.18 (8.58)	0.84 (0.59 to 1.09)	0.46 (0.14 to 0.79)
		Control (n=71)	40.70 (9.29)	42.1 (9.02)	0.15 (0.00 to –0.30)	N/A
	**Behavioral pain coping (FESV)**					
		Intervention (n=78)	30.32 (7.4)	34.59 (7.58)	0.57 (0.36 to 0.78)	0.45 (0.15 to 0.78)
		Control (n=71)	31.76 (7.45)	31.11 (7.83)	–0.08 (–0.25 to 0.08)	N/A

^a^Effect size according to Cohen *d*.

^b^PHQ-ADS: Patient Health Questionnaire Anxiety and Depression Scale.

^c^N/A: not applicable.

^d^PHQ-9: Patient Health Questionnaire-9.

^e^GAD-7: General Anxiety Disorder Scale-7.

^f^PHQ-15: Patient Health Questionnaire-15.

^g^PSS-10: Perceived Stress Scale-10.

^h^HMSE-G-SF: German short form of the Headache Management Self-Efficacy.

^i^Application of behavior change techniques based on the health action process approach stages of change: 1 to 2=nonintenders, 3=intenders, 4 to 5=actors.

^j^HAPA: health action process approach.

^k^Assessment of absenteeism and presenteeism based on Migraine Disability Assessment conducted for a period of 1 month.

^l^MIDAS: Migraine Disability Assessment.

^m^FESV: questionnaire to assess pain management (Fragebogen zur Erfassung der Schmerzverarbeitung).

#### Secondary Outcomes

Regarding secondary outcomes, the ITT LMM analyses demonstrated evidence of treatment effects for depression (t_342_=–3.56; *P*<.001, 95% CI –2.79 to –0.80), somatic symptoms (t_348_=–4.48; *P*<.001, 95% CI –3.35 to –1.31), stress (t_341_=–3.07; *P*=.003, 95% CI –4.25 to –0.94), headache-related self-efficacy (t_342_=4.08; *P*<.001, 95% CI 2.10-5.99), application of BCTs (t_342_=4.82; *P*<.001, 95% CI 0.45-1.07), presenteeism and absenteeism (t_317_=–3.00; *P*=.003, 95% CI –7.96 to –1.68), cognitive pain coping (t_341_=4.96; *P*<.001, 95% CI 3.38-7.79), behavioral coping (t_341_=5.18; *P*<.001, 95% CI 3.11-6.89), and suggestive evidence for anxiety (t_342_=–2.73; *P*=.007, 95% CI –2.50 to –0.40). The PP analyses showed similar results (Table 2). The effect sizes of secondary outcomes between groups after the intervention were medium (eg, depression, anxiety, somatic symptoms, stress, absenteeism and presenteeism, and pain coping) and large (eg, headache-related self-efficacy and application of BCTs). Refer to Table 3 for further details.

#### Predictors

We also explored whether diagnostic status (participants diagnosed with migraine vs participants with other or no headache-related diagnosis), concurrent psychotherapy, concurrent tracking of headaches, and headache-related self-efficacy influenced the pre- and postintervention effects. We did not find evidence of a 3-way interaction among group, time, and predictors. There was no evidence of a difference in the decrease of the PHQ-ADS score between the intervention and waitlist control groups for participants with a diagnosis of migraine (t_338_=–0.81; *P*=.42, 95% CI –5.93 to 2.48), concurrent psychotherapy (t_338_=–0.45; *P*=.65, 95% CI –5.69 to 3.57), concurrent headache tracking (t_338_=–1.92; *P*=.06, 95% CI –6.99 to 0.09), and self-efficacy at baseline (t_338_=–1.50; *P*=.14, 95% CI –0.48 to 0.06).

### Engagement

Table 4 shows the rate of the intended use of the BalanceUP coaching app among participants who started module 1 (n=182), that is, participants from the intervention group (n=110) and participants who started coaching after the waiting time (n=72). As anticipated, the highest dropout rates occurred during module 1 (34/182, 18.7%), with a subsequent decrease in dropout rates during the subsequent modules. Of the 182 participants who began the coaching program with module 1, 118 (64.8%) completed the coaching and thus used it as intended. A visual inspection of the engagement data related to subjective experience revealed that participants who discontinued using the app did not show differences in active participation, internal engagement, perceived benefit, or comprehensibility compared with those who continued using BalanceUP (refer to Multimedia Appendix 6).

**Table 4:** Indicators of engagement: intended use, extent of use, and subjective experience of the BalanceUP app (n=182).

Indicators of engagement and acceptance				All participants who started with module 1^a^
**Intended use of BalanceUP and use data, n (%)**				
	Started coaching (ie, intervention group and waitlist control group after a waiting time of 43 d)			182 (100)
	Ceased interacting during module 1 (dropout)			34 (18.7)
	Ceased interacting during module 2 (dropout)			11 (6)
	Ceased interacting during module 3 (dropout)			6 (3.3)
	Ceased interacting during module 4 (dropout)			4 (2.2)
	Ceased interacting during module 5 (dropout)			2 (1.1)
	Ceased interacting during module 6 (dropout)			1 (0.5)
	Ceased interacting during module 7 (dropout)			6 (3.3)
	Completed outro (intended use)			118 (64.8)
**Extent of use among participants with intended use (n=117-112)**				
	Number of participants who completed all 7 modules, n (%)			101 (86.3)
	Number of days to complete a module, mean (SD)			6.91 (1.50)
	Ratio of reply in conversational turns, mean (SD)			77.84 (3.73)
	Push reminders after 1 h of inactivity, mean (SD)			11.70 (5.99)
	Push reminders after 2 h of inactivity, mean (SD)			8.95 (5.87)
	Push reminders after 1 d of inactivity, mean (SD)			4.14 (3.62)
	Push reminders after 3 d of inactivity, mean (SD)			1.46 (1.66)
	Push reminders after 5 d of inactivity, mean (SD)			0.74 (1.12)
	Email reminders after 10 d of inactivity, mean (SD)			0.34 (0.65)
	Push reminders during survey, mean (SD)			0.75 (0.62)
	Relaxation (audio listened in min), mean (SD)			113.44 (182.52)
**Subjective experience** **among participants with intended use (n=117-112), mean (SD)**				
	Personal involvement during sessions^b^			4.30 (0.55)
	Active participation during sessions^b^			4.27 (0.63)
	Perceived comprehensibility of units^c^			3.87 (0.68)
	Perceived benefit of units^c^			3.87 (0.68)
	Commitment to change^d^			8.15 (5.46)
	Perceived enjoyment^e^			4.13 (0.54)
	Perceived alliance with the chatbot coach^f^			3.94 (0.81)
**Acceptance of the BalanceUP app (n=117-114), mean (SD)**				
	**Self-reported data**			
		Engagement (eg, entertainment and personalization)^g^	3.72 (0.65)	
		Information^g^	4.47 (0.47)	
		Perceived app quality^g^	3.56 (0.77)	
		Perceived impact of the app^g^	4.00 (0.62)	

^a^Participants from the intervention group and waitlist control group who optionally participated after a waiting period.

^b^Measured after every session using 1 item of the Patient Session Evaluation Questionnaire.

^c^Measured randomly at the end of a unit using 1 items of the Patient Session Evaluation Questionnaire.

^d^Measured 3 times during coaching using the question “How committed are you to changing your behavior?”

^e^Measured randomly during coaching by a single item (“Did you enjoy the last unit?”) from technology acceptance research.

^f^Measured 3 times during coaching using the Session Alliance Inventory Scale.

^g^Measured postcoaching intervention via Mobile Application Rating Scale.

We measured engagement among the participants with intended use based on the extent of use (use data) and subjective experience (self-reported data). The use data analysis showed that 86.3% (101/117) of the participants completed all 7 modules, taking an average of 6.9 (SD 1.5) days to work through a module. Participants replied in an average of 77.8% (SD 3.73%) conversational turns. In the event of inactivity, across the entire study period, participants were sent an average of 11.70 (SD 5.99) push notifications for no activity for a 1-hour period and 8.95 (SD 5.87) notifications for no activity for a 2-hour period, with a subsequent decrease in reminders sent for 1, 3, 5, and 10 days of no activity. Participants spent an average of 113.44 (SD 182.51) minutes on relaxation exercises. Refer to Table 4 for further details.

Participants self-reported a mean commitment to change their behavior of 8.15 (SD 5.46). The reported commitment to change behavior significantly increased with time (*F*_2,192_=8.17; *P*<.001) with a medium effect (Cohen *f*=0.29), and evidence of higher commitment toward the end of the coaching (mean 8.17, SD 1.49) than in the middle (mean 7.57, SD 1.55, mean difference 0.60, 95% CI 0.31-0.89; *P*<.001) or beginning of the intervention (mean 7.61, SD 1.57, mean difference 0.56, 95% CI 0.21-0.91; *P*=.002). A linear regression (β=.05, SE 0.42; *P*=.67) with pre- to posttreatment PHQ-ADS changes showed that early reported commitment did not predict before improvement to after improvement of mental well-being. The mean perceived alliance with the CA was 3.94 (SD 0.82). The alliance significantly increased with time (*F*_2,200_=10.66; *P*<.001) with a medium-large effect (Cohen *f*=0.32), with the evidence of alliance becoming higher toward the end of the coaching (mean 4.07, SD 0.83; *P*<.001) than at the beginning (mean 3.83, SD 0.83, mean difference 0.23, 95% CI 0.12-0.34; *P*<.001); refer to Table 5. A linear regression (β=.11, SE 0.80; *P*=.36) with pre- to posttreatment PHQ-ADS change showed that early reported alliance with the CA did not predict pre- to posttreatment improvement of mental well-being.

**Table 5:** Results of a repeated measure ANOVA for commitment to change and perceived alliance with the conversational agent (CA).

Outcome	Start of the intervention^a^, mean (SD)	Midintervention^b^, mean (SD)	End of the intervention^c^, mean (SD)	*P* value	*F* test (*df)*
Commitment to change^d^	7.61 (1.57)	7.57 (1.55)	8.16 (1.49)	<.001	8.17 (2,192)
Perceived alliance with the CA^e^	3.84 (0.85)	4.02 (0.81)	4.07 (0.83)	<.001	10.66 (2,200)

^a^Measured during module 1 (commitment to change) and module 2 (alliance).

^b^Measured during module 3 (commitment to change) and module 4 (alliance).

^c^Measured during module 6.

^d^Measured by the question “How committed are you to changing your behavior” (n=97).

^e^Measured using Session Alliance Inventory Scale (n=101).

Participants with intended use rated the quality of BalanceUP in terms of engagement (eg, personalization and interactivity), information quality (eg, source and credibility), perceived subjective quality (eg, recommend app and pay for app), and perceived impact (eg, knowledge and awareness). The highest-rated subscale was information quality, with a mean of 4.47 (SD 0.47), followed by perceived impact (mean 4.00, SD 0.62), engagement (mean 3.72, SD 0.65), and subjective quality (mean 3.56, SD 0.77).

### Impression of Change and Adverse Events

We found evidence of an improved perceived global impression of change in the Patient Global Impression of Change Scale from module 1 to module 7 (t_101_=8.45; *P*<.001; Cohen *d*=–0.62, 95% CI –0.91 to –0.33), indicating an improvement in perceived general health. Of the participants who reported worsening of their general condition in module 1 (6/142, 4.2%), only 1 participant reported that this was because of the current coaching intervention and provided the following explanation:

Dealing with migraine triggers constant self-monitoring, which may cause slight discomfort and prevents me from feeling completely free from them. However, in the long term, it is worth pursuing education and behavior change.

The remaining 5 participants reported that the worsening was because of other reasons, such as job and social conflicts. In module 7, only 2% (2/102) of the participants reported worsening of their general condition; however, all cited other reasons as the cause.

### Triggers

Participants rated their sensitivity to trigger with a mean of 2.51 (SD 1.09) and avoidance with a mean of 2.32 (SD 1.07), indicating average sensitivity and avoidance compared with a sample of people who have headache [96].

### Qualitative Evaluation

**Figure 7:**
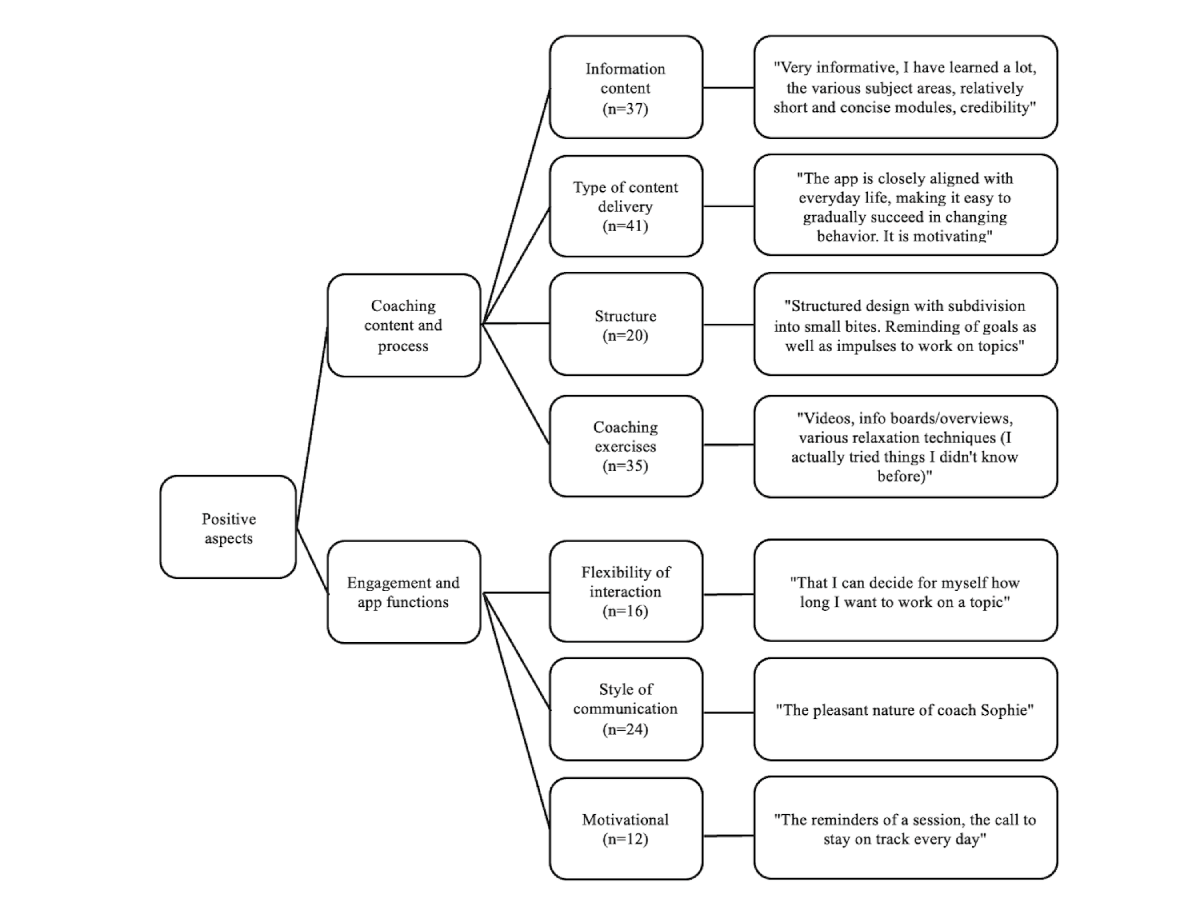
The thematic map illustrates the positive aspects of BalanceUP. Numbers in parentheses indicate the frequency of mentions for each topic.

**Figure 8:**
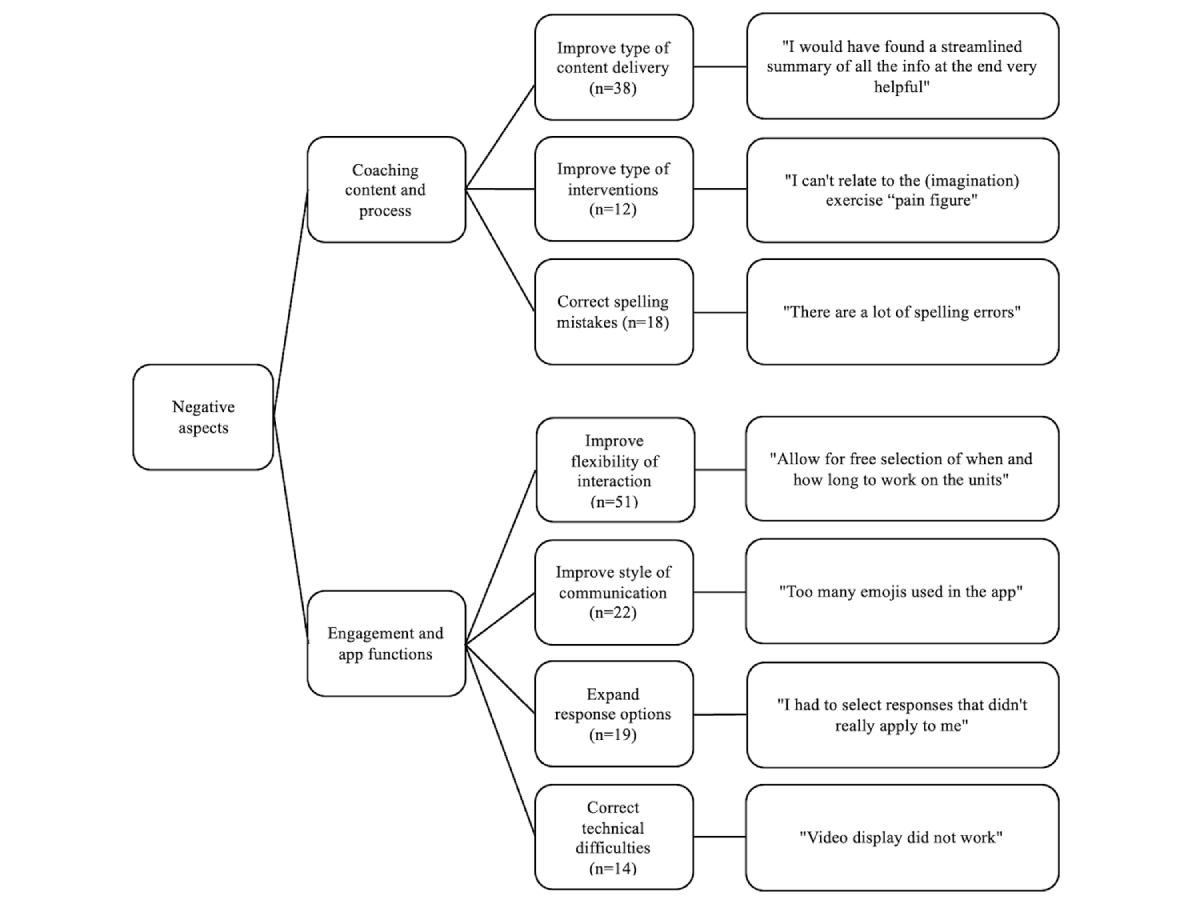
The thematic map illustrates suggestions to improve BalanceUP. Numbers in parentheses indicate the frequency of mentions for each topic.

We further assessed the BalanceUP app's positive (Figure 7) and negative (Figure 8) aspects using qualitative content analysis. We used a deductive approach to identify themes based on insights from our pilot study and the existing literature. When examining the positive aspects, participants expressed a strong appreciation for the extensive and comprehensive information provided, which included various types of exercises and the delivery mode of the intervention. When evaluating negative aspects, participants expressed a desire for more flexibility in their interactions with the CA.

## Discussion

### Principal Findings

This study aimed to describe the development and evaluation of the effectiveness of the BalanceUP app. This is the first RCT of a fully unguided coaching intervention delivered by a rule-based CA to facilitate mental well-being in individuals with headaches. We described the BalanceUP app's evidence-based design and systematic evaluation.

With regard to effectiveness, we found evidence of improved mental well-being, as measured by the PHQ-ADS, in individuals with frequent headaches who received BalanceUP, with a medium to large between-group effect size (Cohen *d*=–0.66). Participants who interacted with BalanceUP experienced a clinically important improvement, reporting, on average, a 3.9 (SD 5.59) point reduction [85] of perceived depression and anxiety symptoms after the intervention. In contrast, participants in the waitlist control group did not show substantial changes in their mental well-being (0.6-point reduction on the PHQ-ADS score). Moreover, we found evidence of reduced anxiety, somatic symptoms, perceived stress, and absenteeism and presenteeism, as well as improved HMSE-G-SF, application of BCTs, and pain coping skills, with effects ranging from medium (Cohen *d*=0.43) to large (Cohen *d*=1.05). Diagnostic status, concurrent psychotherapy, concurrent tracking of headaches, and headache-related self-efficacy did not influence the effects of chatbot coaching.

No notable adverse effects were observed owing to the use of BalanceUP. Among the participants who initiated the coaching by starting the first module, a substantial portion (118/182, 64.8%) successfully adhered to the program. Participants who used BalanceUP as intended established a pronounced working alliance with the CA, which significantly improved with time.

In terms of acceptance, the program’s information content received the highest rating, followed by the perceived impact, engagement, and subjective quality of BalanceUP. Its overall average rating, on a scale of 1 to 5 stars, was 3.91 (SD 0.67), indicating a high level of acceptance. Participants expressed their willingness to recommend the app to individuals who might benefit, with a mean rating of 4.02 (SD 1.05), reflected by the following statement in the uMARS questionnaire: “There are many people I would recommend this app to.”

### Comparison With Prior Work

Evidence on the effectiveness of mobile interventions for headaches is limited, despite their wide availability [47]. Minen et al [44] found that, despite a trend toward mHealth, most studies using electronic behavioral interventions to treat headaches did not use mobile devices. Only 1 single-arm study explored mHealth migraine behavioral therapy but with potential bias owing to missing diary entries. A recent study by Grazzi et al [114] demonstrated the feasibility and effectiveness of an mHealth mindfulness program for chronic migraineurs (headaches for ≥15 d in a month), resulting in a 50% retrospective reduction in migraine days and medication intake.

With regard to internet-based headache treatments, 1 pilot study by Day et al [115] showed improvement in self-efficacy (Cohen *d*=0.82) and pain acceptance (Cohen *d*=0.82), although there was no evidence of headache reduction. An RCT by Bromberg et al [105] assessed a web-based intervention for migraine self-management and coping, revealing improvements in multiple outcomes, including depression, stress, headache self-efficacy, pain catastrophizing, and coping strategies, although without changes in anxiety and disability. Another study on web-based behavioral training for migraine self-management found no difference in headache attack frequency but found evidence for improved migraine self-efficacy and locus of control [116].

This study’s results align with face-to-face behavior change interventions for headaches. Cognitive behavioral therapy, relaxation, or mindfulness interventions have effectively improved the cognitive, behavioral, anxiety, and stress-related aspects associated with headaches [30,117]. Notably, the evaluation of MIMA [72], the basis of our coaching, found limited improvement in headache-related outcomes, except for headache self-efficacy. Furthermore, BalanceUP was extended to address TTH (eg, onset of headache, course of headache, and medication). Although migraine was predominant among participants, we found no evidence that diagnostic status affected treatment outcomes. Tailoring allowed BalanceUP to effectively address both types of headaches.

There was no evidence of the influence of concurrent psychotherapy on coaching effectiveness in this study, which is noteworthy given prior suggestions of potential benefits of combining face-to-face and digital interventions [118,119]. The hypothesized role of headache-related self-efficacy in treatment outcome [120] remained unconfirmed in this study.

The BalanceUP app’s evidence-based design and systematic evaluation contribute to the growing body of evidence on the acceptance and effectiveness of interventions involving CAs in health care. A recent meta-analysis examined 32 RCTs focusing on mental health and CA use [121]. Short-term effects on outcomes such as depressive and generalized anxiety symptoms, quality of life, well-being, and stress were found, with effect sizes ranging from Hedges *g*=0.24 to Hedges *g=*0.62. However, the long-term effects remained unclear. Personalization and empathetic responses emerged as crucial effectiveness facilitators, with longer CA interactions linked to larger effect sizes. These findings align with those of another meta-analysis [122], showing the effectiveness of CAs in outcomes related to lifestyle changes, smoking cessation, substance misuse, and medication adherence. Notably, <50% of the participants reported overall satisfaction with the CA, content likeability, and future use. However, many studies had a pre- and postintervention design or were feasibility trials, indicating the need for further RCTs in this field. Abd-Alrazaq et al [123] evaluated the effectiveness of CAs in 12 clinical and nonclinical RCTs. Weak evidence of reduced depression, stress, or agoraphobia rates, but not improved mental well-being, was found. However, bias, low-quality evidence, small sample size, or contradictory results limited the conclusions, necessitating further high-quality RCTs following the guidelines.

Contextual factors, such as psychological traits, motivation, personal relevance, and attributes of digital behavior change interventions themselves (eg, content, reminders, delivery, support, and personalization) influencing engagement [64]. BalanceUP integrates personalization and tailoring (eg, relevant topic selection and adjustment of interaction length) to empower participants and promote a sense of control and ownership over the coaching process. Emotional support, encouragement, or validation from the CA also expresses empathy, reinforcing participants’ feelings (eg, “This is excellent news, well done, keep on track” and “I am sorry to hear, but setbacks are also part of a change process”). Qualitative feedback confirms program flexibility and suggests that addressing individual preferences can enhance satisfaction and further improve the coaching experience.

In BalanceUP, we observed that 65% of the participants used coaching as intended by completing the outro. In behavioral headache treatment, engagement has yet to be thoroughly assessed in terms of dose and duration; however, earlier studies reported high dropout rates [124].

Consistent with previous research [67,125], participants using BalanceUP established a strong alliance with the CA, which improved significantly with time. This finding aligns with studies conducted in in-person, digital, and group settings [126-128] and with individuals with recurrent headaches [129]. However, contradictory to findings in internet-based therapy studies [130,131], the alliance was unrelated to improvements in participants’ mental well-being. These results suggest that, although the alliance between participants and the CA was established and strengthened throughout the coaching program, other factors may significantly influence treatment effectiveness.

### Limitations and Future Work

This study had several limitations. First, guidelines for trials of behavioral headache treatments [88] recommend using headache frequency as the primary outcome. However, they also urge investigators to use standardized disability, functional status, or quality of life measures. A recent Delphi study by Leudtke et al [132] emphasized the need for outcome measures that truly reflect patients’ experiences. Therefore, the inclusion of functional disability, quality of life, and associated symptoms should be considered in nonpharmacological interventions. BalanceUP aimed to capture the biopsychosocial impact of headaches by addressing various lifestyle factors.

Second, the self-selection of the participants limits the generalizability of our findings and introduces potential self-selection bias. Participants’ particular interest in the subject matter might make them nonrepresentative of the broader population. Caution is needed when extending these findings to a broader context because of the possibility of differing preexisting characteristics.

Third, it is important to acknowledge potential improvements for enhanced interactions in BalanceUP. The current rule-based nature of the CA allowed for the implementation of an evidence-based program. However, participants’ desire for more flexible interactions with the ability to input personal responses was evident. Previous research [133] indicates user preference for a combination of predefined answer options and text input to enhance perceived interactivity. To enhance text processing in BalanceUP, integrating artificial intelligence (AI)–based technology, such as large language models or natural language processing, could be considered. Natural language processing and large language models enable the CA to interpret user inputs more dynamically, yet existing AI-based chatbots struggle with unforeseen user responses [133,134]. Furthermore, ethical aspects related to AI technology should be considered, as they could lead to misjudgments and potential risks. Research has highlighted a lack of transparency in describing the handling of input data and algorithms, affecting the reliability and validity of findings [122,135]. One approach might involve using AI technology for specific tasks (eg, providing content that humans will select or tailor) while maintaining a rule-based approach for other tasks (eg, handling sensitive information and following guidelines), ensuring predictability and preventing harm.

Fourth, it is worth noting that most participants in this study were women (86%), in line with the higher prevalence of headache in women [136]. Approximately half of them had university degrees, consistent with our pilot study [67]. However, the trial’s sociographic distribution may not fully represent the general population. Future studies should seek greater diversity using a more representative sample.

Fifth, a potential limitation is the digital placebo effect [137]. In this unblinded trial, participants might attribute improvements to using an mHealth intervention rather than interventionist ingredients. Expectations and engagement could bias outcomes positively. Future research should carefully design control conditions, considering active control groups or sham interventions [138].

Sixth, participants needing a smartphone introduced a limitation [139]. Although mHealth usability is favored by a substantial proportion of middle-aged individuals with headache in German-speaking countries, the exclusion of smartphone-less individuals may impact generalizability [4]. Ownership rates are high, for example, 92% in Switzerland and 91% in Europe [140].

Seventh, a small number of participants (28/198, 14.1%) in this study encountered technical issues, such as missing audio tones in videos or loss of internet connection. Although technical support was provided to address these problems, it is likely that these technical issues may have had a negative impact on participants’ engagement.

Finally, the primary objective of developing this app was to enhance the mental well-being outcomes of individuals with headache. The app was specifically designed for this research project. Currently, it is freely available for public use. However, the future availability of the app is uncertain because of ongoing support costs and a lack of collaboration with potential providers. We recognize the potential value of making such interventions accessible in the future, and discussions regarding their availability are ongoing. It is essential to note that the university’s role is not to provide the app as a service but to contribute through accompanying research. The sustainability of digital health interventions, particularly with limited public health funding, remains challenging [141,142]. Clear cost-benefit outcomes and accountability strategies should be addressed in future research.

### Conclusions

This study represents the first empirical evaluation of an evidence-based and CA-delivered coaching intervention (BalanceUP) designed to promote a healthy lifestyle among individuals with headaches. The findings provide evidence of the positive impact of BalanceUP on participants’ mental well-being, as indicated by improvements in depression and anxiety symptoms. The establishment of a strong alliance between participants and the CA, along with their high commitment to the program, further reinforces the potential effectiveness of this intervention. The scalability and accessibility of automated coaching through a CA highlight its potential as an engaging and effective tool for behavior change. Further research is needed to explore the long-term effects, intensity, and duration of delivery of coaching interventions for lifestyle modifications in the health sector. Therefore, the applicability of mHealth interventions in diverse populations should be investigated. Understanding these aspects will contribute to the development of effective and inclusive interventions that promote positive health outcomes.

## Appendix

Multimedia Appendix 1 Overview of the coaching intervention.

Multimedia Appendix 2 Intervention flow.

Multimedia Appendix 3 Behavior change techniques implemented.

Multimedia Appendix 4 Aspects of engagement.

Multimedia Appendix 5 Outcomes and time points.

Multimedia Appendix 6 Engagement with subjective experience dropout versus intended use.

Multimedia Appendix 7 CONSORT-EHEALTH Checklist.

## Abbreviations

AI: artificial intelligence
BCT: behavior change technique
CA: conversational agent
CONSORT: Consolidated Standards of Reporting Trials
GAD-7: General Anxiety Disorder Scale-7
HMSE-G-SF: German short form of the Headache Management Self-Efficacy
ITT: intention-to-treat
LMM: linear mixed model
mHealth: mobile health
MIMA: Cognitive-Behavioral Therapy for Migraine Management (Kognitiv-verhaltenstherapeutisches Migränemanagement)
PHQ-15: Patient Health Questionnaire-15
PHQ-9: Patient Health Questionnaire-9
PHQ-ADS: Patient Health Questionnaire Anxiety and Depression Scale
PP: per-protocol
RCT: randomized controlled trial
TTH: tension-type headache
uMARS: translated version of the Mobile App Rating Scale

## Data Availability

The data sets generated and analyzed during this study are available from the corresponding author upon reasonable request.
