# Supplementary material for: Development and Evaluation of a Smartphone-Based Chatbot Coach to Facilitate a Balanced Lifestyle in Individuals With Headaches (BalanceUP App): Randomized Controlled Trial
Source: J Med Internet Res. 2024 Jan 24;26:e50132. doi: 10.2196/50132 (PMC10851123; doi:10.2196/50132)
Supplement: Multimedia Appendix 1 [file jmir_v26i1e50132_app1.pdf]

Appendix 1. Overview of coaching with core themes and activities (translated from German).

Module	Unit	Topics, exercises and tasks[a]
Onboarding		
	0	• Introduction of the digital coach, coaching, and the study<br>• Set coaching intention and define an object to reflect/remember the intention<br>• Watch a video about the aim of the study and the aim of the coaching
1 Psychoeducation		
	1	• Browse different models about how headaches can develop (e.g.: functional condition model, trigger model)<br>• Watch educational videos (e.g.: migraine development)<br>• Exercise “mindful sprint” to reflect on crucial topics of the lesson
	2	• Browse different symptoms of headaches
	3	• Browse the course of headaches (e.g. TTH)<br>• Listen to relaxation exercise (body scan)<br>• Plan the integration into the daily routine and anticipate obstacles<br>• Watch an educational video on the topic and complete the optional quiz
2 Relaxation		
	4	• Browse different relaxation activities and how they work<br>• Watch educational videos (relaxation and headaches)<br>• Anticipate difficulties of relaxation and strategies to cope<br>• Perform meditation exercise
	5	• Learn about different types of mindfulness strategies (e.g. formal and informal practice) and how they can be applied<br>• Plan to adopt mindfulness exercises between coaching sessions (e.g. sensing fragrance in a flower store)<br>• Exercise “mindful sprint” to reflect on crucial topics of the lesson
	6	• Introduction on the concept of acceptance and headaches<br>• Brows different breathing exercises<br>• Learn and experiment with different breathing exercises in different contexts
	7	• Emphasize the importance of physical activity in relation to headache<br>• Browsing different aspects of physical activity (e.g.: type of activity, intensity, motivation)<br>• Planning to integrate physical activity into the daily routine<br>• Practicing imagination exercise
3 Balanced lifestyle		
	8	• Discuss the association between your lifestyle and headaches and watch an educational video<br>• Learn about the importance of regular lifestyle and sudden changes in stress and recovery<br>• Set goals towards a balanced lifestyle (e.g., regular recovery) and plan implementation into the daily routine
	9	• Reflect on personal values, and reflect on domains of life in relation to resources (e.g. family, work, etc.)<br>• Anticipating optimization of recourses and efforts in different life domains<br>• Exercise “mindful sprint” to reflect on crucial topics of the lesson
	10	• Familiarize yourself with the concept of shielding stimuli and watch an educational video

		• Browse different strategies to keep away negative stimuli (e.g. retreat, breathing, imagination)
		• Planning to adopt imagination (stimuli shield) in a relevant situation
	11	• Emphasize importance of regular sleep habits
		• Reflect own sleeping habits and brows healthy sleep habits (e.g.: napping, rituals)
4 Cope with fear of headache attack		
	12	• Introduce the function of fear and its impact on behavior and vicious circle
		• Step-by-step guidance for sustainable integration of a previously set goal
	13	• In-dept discussion of vicious circle of fear resulting in dysfunctional behavior, watch educational video
		• Consider a scenario with experiencing fear of headache attack and reflect related emotions, thought patterns, and dysfunctional behavior by guided behavior analyses
	14	• Learn about alternative thoughts and coping styles (functional behavior)
		• Guided reflection of options (helpful thoughts) to coping with fear
		• Prepare a “plan B” in case of a headache
		• Familiarize yourself with the concept of acceptance
		• Listen imagination exercise (focus on own resources)
5 Coping with headaches		
	15	• Browse medications for acute headaches, learn about applications, positive and negative aspects of medication application (e.g.: regular intake, medication overuse)
		• Commit to the use of an alternative strategy for the case of mild headaches (e.g. heat or cold pack, imagination)
		• Watch video about using ice friction for mild headaches
	16	• Browse different medications, their use, and positive and negative aspects
		• Review the optimization of resources and efforts in different life domains
	17	• Familiarize with the concept of avoidance vs endurance and analyze own behavioral patterns
		• Reflect on own priorities in life (e.g. work, social activities)
		• Consider if and how to communicate in case of headache related absence
		• Watch educational video about different types of headache-specific massages (e.g. face, neck, shoulder)
	18	• Reflect on your functional strategies to cope with headaches
		• Discuss different options of behavior in case of headaches (e.g.: avoid stimuli, fresh air, distraction)
		• Review your behavior with regard to endurance and plan for breaks and retreats as appropriate
		• Listen to imagination exercice (perception of pain)
		• Exercise “mindful sprint” to reflect on crucial topics of lesson
6 Headache trigger		
	19	• Introduction into topic of trigger and how they are related to headaches
		• Find out about most common triggers
		• Note that triggers are often not reliable and do not always trigger a headache attack, avoidance is not always effective
	20	• Analyze personal trigger (HTSAQ[b]) in terms of response and avoidance

	21	• Browse different strategies in dealing with triggers (e.g.: avoidance, habituation)<br>• Reflect on own triggers in relation to response and avoidance<br>• Anticipate alternative strategies in dealing with triggers<br>• Plan imagination exercise (perception and change of perception)
	22	• Familiarize yourself with different recommendations on specific triggers (e.g.: stress, sleep, avoidance)
7 Stress management		
	23	• Browse different aspects and symptoms of stress (e.g.: stress reaction)<br>• Understand the interaction of stressors, stress enhancer and stress reaction
	24	• Understand relation of stress and headaches and identify stressors<br>• Guided instruction to identify own reaction to stress: physical, behavioral, emotional, and thought patterns<br>• Watch a video clip about cervical spine exercises
	25	• Understand 3 dimensions of stress coping (situational, mental, regenerative)<br>• Understand different strategies to deal with stress:<br>o Define a situation where boundaries should be set<br>o Identify own social network as recourse and plan strategies to cultivate social network<br>o Understand the concept of inner distance to stressors
	26	• Importance of recovery: strengthen awareness of signs of stress and set intention to focus on these signs within next weeks<br>• Quiz on stress competencies
Between modules		
		• Anticipate difficulties of relaxation and strategies to cope with difficulties
Outro		
		• Appreciate your achievement<br>• Reflect on personal goal achievement and plan to keep the goals in mind (write a letter to you)

[a]Topics and tasks can be selected as appropriate.
[b]Headache Triggers Sensitivity and Avoidance Questionnaire, German short version.
