# Supplementary figures and images for: Development and Evaluation of a Smartphone-Based Chatbot Coach to Facilitate a Balanced Lifestyle in Individuals With Headaches (BalanceUP App): Randomized Controlled Trial
Source: J Med Internet Res. 2024 Jan 24;26:e50132. doi: 10.2196/50132 (PMC10851123; doi:10.2196/50132)

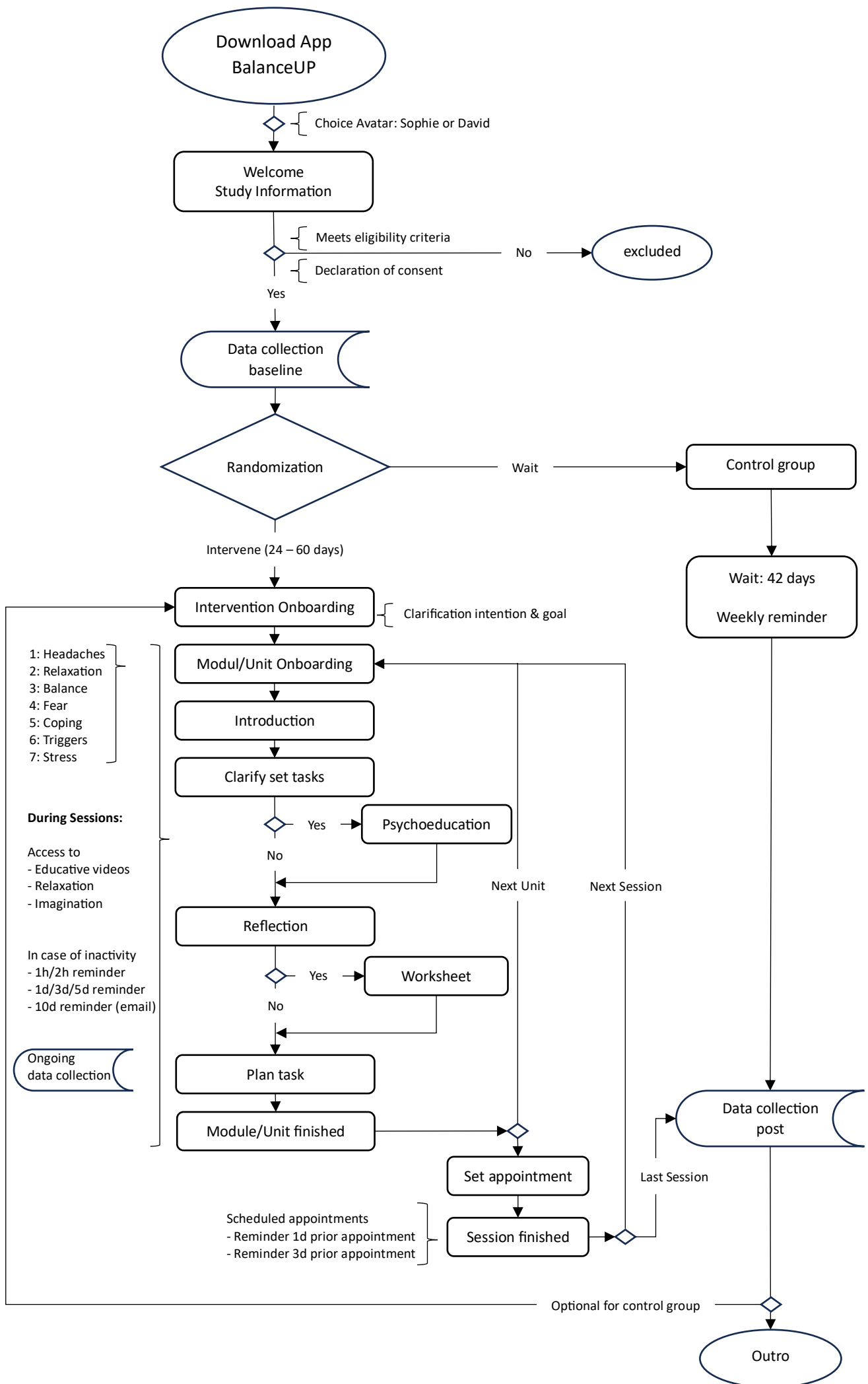

Supplement: Multimedia Appendix 2 [file jmir_v26i1e50132_app2.pdf]
