# Supplementary material for: Development and Evaluation of a Smartphone-Based Chatbot Coach to Facilitate a Balanced Lifestyle in Individuals With Headaches (BalanceUP App): Randomized Controlled Trial
Source: J Med Internet Res. 2024 Jan 24;26:e50132. doi: 10.2196/50132 (PMC10851123; doi:10.2196/50132)
Supplement: Multimedia Appendix 3 [file jmir_v26i1e50132_app3.pdf]

Appendix 6. List of BCTs[a] applied in BalanceUP.

No	Label	Definition	Examples
**1. Goals and planning**			
1.1	Goal setting (behavior)	Set or agree on a goal defined in terms of the behavior to be achieved	Agree on a mindfulness-based activity during the next week
1.2	Problem solving	Analyze, or prompt the person to analyze, factors influencing the behavior and generate or select strategies that include overcoming barriers and/or increasing facilitators	Analyze barriers to adopt relaxation and reflect how to overcome these
1.3	Goal setting (outcome)	Set or agree on a goal defined in terms of a positive outcome of wanted behavior	Set an intervention intention (e.g. get to know more information, adopt relaxation, reduce medication)
1.4	Action planning	Prompt detailed planning of performance of the behavior (must include at least one of context, frequency, duration, and intensity)	Do sports/be active for at least 3 times 30 min per week, practice with friends, make a calendar entry
1.5	Review behavior goal(s)	Review behavior goal(s) jointly with the person and consider modifying goal(s) or behavior change strategy considering achievement	Examine how well relaxation practice went and if help/advice is needed for further practice
1.6	Discrepancy between current behavior and goal	Draw attention to discrepancies between a person's current behavior (in terms of the form, frequency, duration, or intensity of that behavior) and the person's previously set outcome goals, behavioral goals or action plans	Point out to the participant that the planned mindfulness activities $was not reached
1.7	Commitment	Ask the person to affirm or reaffirm statements indicating commitment to change the behavior	Before and after action planning, ask the participant to judge on own commitment
**2. Feedback and monitoring**			
2.2	Feedback on behavior	Monitor and provide informative or evaluative feedback on performance of the behavior	Inform the participant of minutes of relaxation and imagination exercises performed so far
2.3	Self-monitoring of behavior	Establish a method for the person to monitor and record their behavior(s) as part of a behavior change strategy	Ask the participant to reflect own behavior in terms of fear (vicious cycle) with the help of a worksheet
**3. Social support**			
3.1	Social support (unspecified)	Advise on, arrange or provide social support	Constant encouragement by the coach for applied relaxation
3.2	Social support (practical)	Advise on, arrange, or provide practical help (e.g. friends,	Set up a challenge with a friend or family member to practice relaxation

		relatives,) for performance of the behavior	
3.3	Social support (emotional)	Advise on, arrange, or provide emotional social support (e.g. friends, relatives) for performance of the behavior	Reflect own social network and plan concrete actions to consider social network for resources in stressful times
**4. Shaping knowledge**			
4.1	Instruction on how to perform a behavior	Advise or agree on how to perform the behavior (includes 'Skills training')	Elaborated instructions on how to perform relaxation
4.2	Information about antecedents	Provide information about antecedents that reliably predict performance of the behavior	Keep track of potential triggers or situations that may lead to headaches
4.3	Re-attribution	Elicit perceived causes of behavior and suggest alternative explanations	If a person avoids certain triggers because he/she believes it causes headaches, suggest checking if this really happens as it might not be the case
4.4	Behavioral experiments	Advise on how to identify and test hypotheses about the behavior, its causes and consequences, by collecting and interpreting data	Explore different triggers and see if it leads to headaches (experiment) and keep track of situations and headaches
**5. Natural consequences**			
5.1	Information about health consequences	Provide information about health consequences of performing the behavior	Explain that taking medication more than 10 times per month can cause headaches
5.4	Monitoring of emotional consequences	Prompt assessment of feelings after attempts at performing the behavior	Reflect which feelings came into mind when having troubles relaxing
5.6	Information about emotional consequences	Provide information about emotional consequences of performing the behavior	Inform that relaxation increases happiness and life satisfaction
**6. Comparison of behavior**			
6.1	Demonstration of the behavior	Provide an observable sample of the performance of the behavior, directly in person or indirectly e.g. via film, pictures, for the person to aspire to or imitate	By means of video, demonstrate to participants how to perform the imagination exercise "sensory isolation/stimulus shielding"
6.2	Social comparison	Draw attention to others' performance to allow comparison with the person's own performance	Inform participants about their sensitivity to potential triggers and their tendency to avoid triggers, in comparison to other headache sufferers.
**7. Associations**			
7.1	Prompts/cues	Introduce or define environmental or social stimulus with the purpose of	Send push notifications on participants smartphone to remind them to perform relaxation

		prompting or cueing the behavior	
7.6	Satiation	Advise or arrange repeated exposure to a stimulus that reduces or extinguishes a drive for the unwanted behavior	Advise participants to gradually expose themselves to triggers to reduce their impact on headaches
7.7	Exposure	Provide systematic confrontation with a feared stimulus to reduce the response to a later encounter	Agree with participants to go to the cinema or a shopping center, even if a headache may occur afterward
**8. Repetition and substitution**			
8.1	Behavioral practice/ rehearsal	Prompt practice or rehearsal of the performance of the behavior one or more times in a context	Encourage participants to practice relaxation techniques in noisy places as well, this will help them incorporate these techniques into their daily routines more easily, instead of just relying on quiet environments
8.2	Behavior substitution	Prompt substitution of the unwanted behavior with a wanted or neutral behavior	Suggest that participants try using their imagination to alleviate mild headaches, rather than immediately resorting to medication
8.3	Habit formation	Prompt rehearsal and repetition of the behavior in the same context repeatedly so that the context elicits the behavior	Prompt participants to regularly to perform relaxation
8.4	Habit reversal	Prompt rehearsal and repetition of an alternative behavior to replace an unwanted habitual behavior	Encourage participants to use alternative strategies to alleviate mild headaches instead of relying solely on medication
8.6	Generalization of a target behavior	Advise to perform the wanted behavior, which is already performed in a particular situation, in another situation	Advise participants to initially practice relaxation techniques in a quiet environment and gradually transition to practicing them in noisy or crowded places
8.7	Graded tasks	Set easy-to-perform tasks, making them increasingly difficult, but achievable, until behavior is performed	Encourage participants to start practicing relaxation techniques for 5 minutes, and then gradually increase the duration if they have successfully achieved the 5-minute mark
**9. Comparison of outcomes**			
9.1	Credible source	Present verbal or visual communication from a credible source in favor of or against the behavior	Present information based on the latest scientific knowledge and research, in collaboration with experts such as scientists, doctors, and psychologists, and following established guidelines
9.2	Comparative imagining of future outcomes	Prompt or advise the imagining and comparing of future outcomes of changed versus unchanged behavior	Advice the participant to reflect possible outcomes following avoidance or endurance
**10. Reward and threat**			

10.3	Non-specific reward	Arrange delivery of a reward if and only if there has been effort and/or progress in performing the behavior	Provision of a certificate only if the coaching was completed within a predefined number of days
10.4	Social reward	Arrange verbal or non-verbal reward if and only if there has been effort and/or progress in performing the behavior	Positive reinforcement of participants if the assigned exercises have been completed
10.6	Non-specific incentive	Inform that a reward will be delivered if and only if there has been effort and/or progress in performing the behavior	Instruct participants on how to consistently adopt alternative behaviors and, if successful, arrange for a pre-specified reward
10.7	Self-incentive	Plan to reward self in future if and only if there has been effort and/or progress in performing the behavior	Suggestion by the coach to reward oneself for doing relaxation regularly
**11. Regulation**			
11.1	Pharmacological support	Provide, or encourage the use of or adherence to, drugs to facilitate behavior change	Explain the use of different medications and recommend taking antiemetics in combination with pain medication in case of a migraine
11.2	Reduce negative emotions	Advise on ways of reducing negative emotions to facilitate performance of the behavior	Advise on the use of concept of acceptance to reduce anxiety about a potential migraine attack
11.3	Conserving mental resources	Advise on ways of minimizing demands on mental resources to facilitate behavior change	Advise to use a systematic training plan for trigger habituation with gradual dose increase to keep track of the trigger
**12. Antecedents**			
21.1	Restructuring the physical environment	Change, or advise to change the physical environment in order to facilitate performance of the wanted behavior or create barriers to the unwanted behavior (other than prompts/cues, rewards and punishments)	Advise to prepare sports equipment the night before – to have it ready when leaving the house
12.2	Restructuring the social environment	Change, or advise to change the social environment in order to facilitate performance of the wanted behavior or create barriers to the unwanted behavior	Advise a person to withdraw from environments with excessive stimulation when a headache develops
12.3	Avoidance/reducing exposure to cues for the behavior	Advise on how to avoid exposure to specific social and contextual/physical cues for the behavior, including changing daily or weekly routines	Suggest individuals who suffer from migraines to maintain regular sleep habits, as changes in sleep patterns are often associated with triggering migraines
12.4	Distraction	Advise or arrange to use an alternative focus for attention to avoid triggers for unwanted behavior	Suggest focusing on specific breathing techniques whenever stress arises to counteract on headache

12.6	Body changes	Alter body structure, functioning or support directly to facilitate behavior change	Prompt relaxation training
**13. Identity**			
13.2	Framing/reframing	Suggest the deliberate adoption of a perspective or new perspective on behavior in order to change cognitions or emotions about performing the behavior	Unconditional perseverance should be avoided at the onset of headaches. Taking a break should not be interpreted as weakness
13.3	Incompatible beliefs	Draw attention to discrepancies between current or past behavior and self-image, in order to create discomfort	Draw attention to a person's behavior in terms of endurance and avoidance and their self-perception of a balanced behavior
13.4	Valued self-identity	Advise the person to write or complete rating scales about a cherished value or personal strength as a means of affirming the person's identity as part of a behavior change strategy	Recommend that individuals assess their current understanding of their own stress reactions before receiving further information on stress management
**14. Scheduled consequences**			
14.8	Reward alternative behavior	Arrange reward for performance of an alternative to the unwanted behavior	Arrange with a person to reward themselves for being physically more active (eg, to use stairs)
**15. Self-belief**			
15.1	Verbal persuasion about capability	Tell the person that they can successfully perform the wanted behavior, arguing against self-doubts and asserting that they can and will succeed	Tell the person that they can successfully adopt relaxation/imagination, even if it seems very hard to focus from time to time
15.2	Mental rehearsal of successful performance	Advise to practice imagining performing the behavior successfully in relevant contexts	Encourage the person to visualize positive outcomes that can result from engaging in imaginative practices
15.3	Focus on past success	Advise to think about or list previous successes in performing the behavior (or parts of it)	Advise to imagine situations in which the headache did not occur as expected, and one was successfully armed against negative thoughts
15.4	Self-talk	Prompt positive self-talk (aloud or silently) before and during the behavior	Encourage individuals to verbally affirm to themselves that they are capable of overcoming obstacles that hinder their participation in relaxation activities

[a]BCT: behavior change technique taxonomy (v1), Michie et al., 2013.
