# Supplementary material for: Development and Evaluation of a Smartphone-Based Chatbot Coach to Facilitate a Balanced Lifestyle in Individuals With Headaches (BalanceUP App): Randomized Controlled Trial
Source: J Med Internet Res. 2024 Jan 24;26:e50132. doi: 10.2196/50132 (PMC10851123; doi:10.2196/50132)
Supplement: Multimedia Appendix 4 [file jmir_v26i1e50132_app4.pdf]

Appendix 7. Aspects of engagement focused on in BalanceUP.

**Tailoring/Personalization**	
Method	Example
• Selection of a CA	• Select a male (David) or female (Sophie) CA, see Figure 1A
• Set personalized goals related to behavior change	• Specify a mindfulness-based activity for the time until the next session
• Option to skip or select certain topics of units based on individual interests, health literacy or diagnosis	• Only work through materials that are related to migraine, but skip those related to TTH
• Additional working materials	• Access worksheets (eg, behavior in case of headaches) for reflective exercises to support personal development
• Appointment with the CA to ensure that coaching is delivered at the optimal time for each participant [80]	• Only complete two units of a module and continue another day with the remaining units
**Personality of the CA**	
• Appearance	• Age reflecting the target population [81]
• Personality	• Approachable, empathetic, and coach-like [69]
• Communication style	• Supportive and social talk to foster a sense of connection between the user and the CA [50,57,82]
**Reminder for scheduled appointments and related tasks[a]**	
• 1 day prior to appointment	• $greeting $participantName, Looking forward to our meeting tomorrow 😄<br>• $greeting $participantName, Tomorrow we will find out whether and what triggers play a role with you. Bye 👋, $coachName
• 3 days prior to appointment	• $greeting $participantName, Moving on in three days 😄...you're almost there...wow🙌<br>• $greeting $participantName, Have you been able to use an alternative strategy for mild headaches such as ☕ or retreating to a quiet place? See you soon, $coachName

**Reminder for inactivity during a coaching unit[a]**	
Timepoint	Examples
• 1 hour	• $participant Name, It was probably inconvenient before. How does it look now? Do you want to continue chatting?"
• 2 hours	• $participantName, It's time to answer $coachName....
• 1 day	• $participantName, We were interrupted yesterday. Are we still going to finish our session today?

Timepoint	Examples
• 3 days	• $participantName, It's good that you are taking timeouts. It’s now been three days since your last session. When you are ready, pick up the thread again with $coachName!
• 5 days	• $participantName, It’s been a few days since $coachName last heard from you. You must have been busy, but that's ok. Please continue with the coaching, $coachName is waiting for you! Or are you having technical problems? If so, please contact balanceup.pi@zhaw.ch.
• 10 days	• Hello $participantName! It's me, your coach $coachName. I haven't heard from you in 10 days. Are you having technical issues with the BalanceUP app? If so, you can contact my team via balanceup.pi@zhaw.ch by simply replying to this email. Maybe you were also on vacation or otherwise busy. I understand. Do you still intend to finish the coaching? Please get in touch.
**Reminder for inactivity during data collection**	
Timepoint	Examples
• 10 min	• $participantName, Something seems to have come up for you....please complete the questionnaire so that coaching can start soon!
• 30 min	• $participantName, Unfortunately, you haven’t completed the questionnaire yet. Please answer all questions; only then can your data be evaluated. Thank you very much!

[a]If a user does not respond to a reminder, a maximum of 6 reminders will be sent. Reminders are reset after the completion of a coaching unit (ie, the escalation process starts anew with each new coaching unit).
