# Supplementary material for: Development and Evaluation of a Smartphone-Based Chatbot Coach to Facilitate a Balanced Lifestyle in Individuals With Headaches (BalanceUP App): Randomized Controlled Trial
Source: J Med Internet Res. 2024 Jan 24;26:e50132. doi: 10.2196/50132 (PMC10851123; doi:10.2196/50132)
Supplement: Multimedia Appendix 5 [file jmir_v26i1e50132_app5.pdf]

Appendix 9. Self-reported outcomes and timepoints.

Time of assessments	T1[a]	Btw.[b]	T2[c]
**Screening**			
Inclusion criteria	X		
Study information	X		
Informed consent (digital)	X		
**Demographics**			
Participant characteristics	X		
Headache related	X		
**Primary outcome**			
Mental well-being PHQ-ADS (PHQ-9 & GAD-7)	X		X
**Secondary outcomes**			
Psychosomatic symptoms PHQ-15	X		X
Perceived Stress Scale PSS-10	X		X
Headache Management Self-Efficacy Scale HMSE	X		X
Stage of change HAPA	X		X
Absenteeism/Presentism MIDAS[d]	X		X
Pain Coping FESV	X		X
**Engagement and Acceptance**			
Session Alliance Inventory SAI		X[e]	
Trigger HTSAQ-G-SF		X[f]	
Engagement to sessions GTS-P		X[g]	
Session Evaluation GTS-P		X[h]	
Commitment to change behavior		X[i]	
Feasibility and Acceptance uMars			X
**Adverse events**			
Patients' Global Impression of Change PGIC		X[j]	
Adverse events		X[j]	

[a]Baseline.
[b]Between pre-treatment and post-treatment.
[c]Primary endpoint. Intervene: Between days 24 to 60. Wait: Day 42, acceptance between days 66 to in case of optional coaching participation.
[d] Reduced number of items.
[e]Measured at the end of Modules 2, 4, and 6.
[f]Measured in Module 6 (optional).
[g]Measured after ending a session.
[h]Measured randomized after completion of a unit.
[i]Measured during onboarding, and in Modules 3 and 6.
[j]Measured in Modules 1 and 7.
