# Supplementary material for: Development and Evaluation of a Smartphone-Based Chatbot Coach to Facilitate a Balanced Lifestyle in Individuals With Headaches (BalanceUP App): Randomized Controlled Trial
Source: J Med Internet Res. 2024 Jan 24;26:e50132. doi: 10.2196/50132 (PMC10851123; doi:10.2196/50132)
Supplement: Multimedia Appendix 6 [file jmir_v26i1e50132_app6.pdf]

Appendix

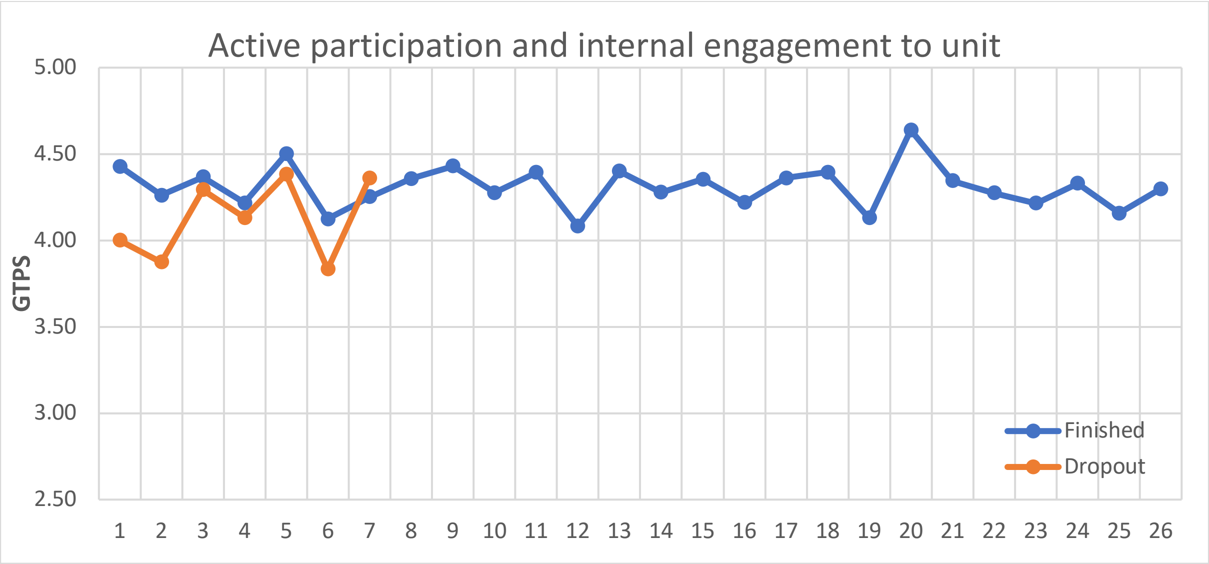

Note: Mean value of self-reported value of two items of the Patient Session Evaluation Questionnaire (GTS-P) regarding active participation and internal engagement to a unit, measured after every session. Finished: n=31-113, Dropout: n=12-35.

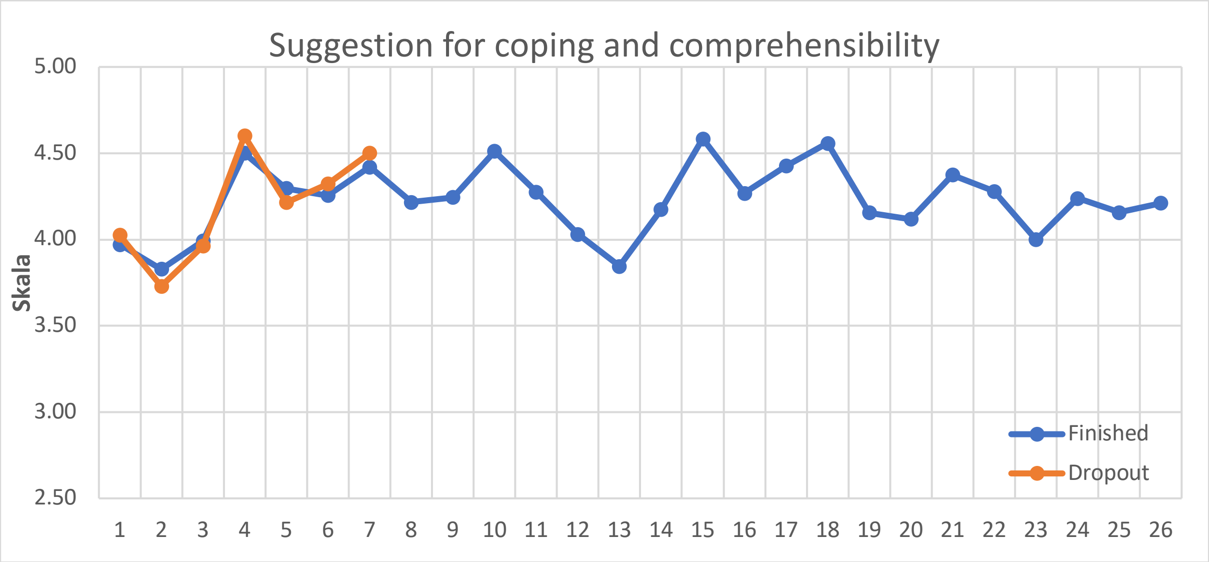

Note: Mean value of self-reported value of two items of the Patient Session Evaluation Questionnaire (GTS-P) regarding perceived value of suggested coping strategies and comprehensibility of a unit, measured randomly after every unit. Finished: n=42-64, Dropout: n=7-20.
